# Regulating Enzyme Activity via Microaggregates Mediated by Phase Separation

**DOI:** 10.1002/advs.202509209

**Published:** 2025-07-23

**Authors:** Yang Wang, Juzheng Yuan, Niu Dai, Yanlin Ji, Wenguang Yang, Xiao Li, Siqi Yan, Jin Yan

**Affiliations:** ^1^ Department of Hepatology The Second Affiliated Hospital of Xi'an Jiaotong University Xi'an 710004 P. R. China; ^2^ Department of Tumor and Immunology in precision medical institute, Western China Science and Technology Innovation Port The Second Affiliated Hospital of Xi'an Jiaotong University Xi'an 710004 P. R. China; ^3^ Department of Hepatobiliary Surgery, Xijing Hospital The Fourth Military Medical University Xi'an 710032 P. R. China; ^4^ Department of General Surgery, Xijing Hospital The Fourth Military Medical University Xi'an 710032 P. R. China; ^5^ Department of Medical Oncology and Department of Talent Highland The First Affiliated Hospital of Xi'an Jiaotong University Xi'an 710061 P. R. China

**Keywords:** chiral peptide, enzyme sequestration, microaggregates, phase separation, postoperative pancreatic fistula

## Abstract

Liquid–solid phase separation (LSPS) is a biomolecular segregation process forming solid‐like aggregates that encapsulate and concentrate specific biomolecules, thus profoundly influencing biochemical reactions. Building on this phenomenon, we developed an enzyme‐regulation strategy inducing LSPS of target enzymes to isolate them from substrates. As proof of concept, we created a novel D‐Peptide mediated Microaggregate Degradation named DPMD to capture trypsin and chymotrypsin in the seroperitoneum during postoperative pancreatic fistula (POPF). DPMD is designed with cationic and hydrophobic residues and a β‐sheet‐forming motif for specific binding. The core principle is an entropy‐driven peptide self‐assembly that sequesters these proteases from the extracellular environment. Captured enzymes form intricate peptide–enzyme microaggregates, effectively segregating them from substrates. These microaggregates are readily internalized and cleared by macrophages, likely via macropinocytosis. In rat POPF models, DPMD significantly reduced pancreatic fluid leakage and inflammatory markers, improved survival, and outperformed a conventional L‐peptide control. Toxicity evaluations showed DPMD was well tolerated even at doses beyond therapeutic levels, underscoring its favorable safety profile. In summary, this study demonstrates a significant translational advance in POPF treatment and expands our understanding of pathogenic enzyme sequestration via LSPS, potentially opening new therapeutic avenues for a range of enzyme‐mediated diseases.

## Graphical abstract

An enzyme‐sequestration strategy for combating postoperative pancreatic fistula (POPF) is developed, employing a D‐peptide called DPMD to induce liquid–solid phase separation (LSPS) of pancreatic proteases. This LSPS‐driven microaggregate approach effectively isolates trypsin and chymotrypsin from their substrates, thereby reducing pancreatic fluid leakage and inflammation, and improving survival in a rat POPF model.

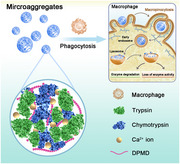

## 1 Introduction

Liquid–solid phase separation (LSPS) is a fundamental process in cellular biology, wherein certain intrinsically disordered proteins (IDPs) or peptides can undergo irreversible aggregation to form solid microaggregates.^[^ ^1^ ^]^ These solid microaggregates have attracted considerable attention because of their exceptional capability to encapsulate and concentrate biomolecules, thereby exerting a profound impact on biochemical reactions and modulating protein function both within and outside the cell.^[^ ^2^ ^]^

The occurrence of postoperative pancreatic fistula (POPF), a severe complication that is the leading cause of death following pancreatic surgery, is characterized by the activation and release of digestive enzymes such as trypsin and chymotrypsin into the surrounding tissues, resulting in autodigestion and inflammation.^[^ ^3^ ^]^ Although traditional approaches to managing POPF involve the use of enzyme inhibitors and sealing materials to prevent enzyme leakage, these methods have limitations, including incomplete inhibition of enzymatic activity, potential adverse effects from enzyme inhibitors, and wound healing disorders caused by sealing materials.^[^ ^4^ ^]^ Consequently, they have not significantly reduced the incidence or mortality associated with POPF.^[^ ^4^, ^5^ ^]^ Inspired by the remarkable ability of peptide and/or protein microaggregates to encapsulate and concentrate biomolecules, it can be hypothesized that LSPS‐mediated microaggregates could potentially be harnessed to manage POPF by sequestering these digestive enzymes within peptide microaggregates, thereby modulating their activity and preventing tissue damage. By leveraging the irreversible phase transition to concentrate enzymes, this LSPS‐based approach provides a more precise and efficient means of enzyme regulation compared to traditional inhibitors. This method not only minimizes systemic side effects but also enhances therapeutic efficacy.

To accomplish this, we have successfully developed a D‐ Peptide mediated Microaggregate Degradation called DPMD, which exhibits an affinity for trypsin and chymotrypsin and coordinates the assembly of intricate peptide‐enzyme microaggregates via LSPS. The peptide sequence DPMD (Ac‐(D‐RADA)_4_‐CONH_2_) was purposefully designed based on the well‐characterized (RADA)_4_ self‐assembling motif. This alternating arginine (R) and aspartic acid (D) sequence is known to form β‐sheet nanofibers that can assemble into higher‐order structures, making it an excellent scaffold for molecular sequestration. We chose the D‐amino acid configuration to ensure stability against proteolysis, given the protease‐rich environment of postoperative pancreatic fluid. Importantly, the (RADA)_4_ motif provides multiple charged residues that can interact with serine proteases such as trypsin and chymotrypsin – for example, arginine side chains can engage negatively charged pockets on the enzyme surface, while aspartate residues can form salt bridges with positively charged regions of the enzymes. These features were hypothesized to confer a degree of specificity: DPMD was expected to preferentially bind and aggregate trypsin and chymotrypsin due to complementary electrostatic and hydrogen‐bond interactions, a hypothesis supported by our experimental data. This rational design approach differentiates DPMD from peptides identified through random screening, grounding its specificity and effectiveness in known molecular interaction principles. This sequestration effectively isolates the enzymes from their substrates, thus proficiently inhibiting their activity and preventing further damage to pancreatic tissue. Furthermore, these peptide‐enzyme microaggregates are designed to be internalized by macrophages via macropinocytosis, facilitating their clearance from the inflamed area. This dual mechanism not only halts enzymatic activity but also facilitates the efficient removal of enzymes from sites of inflammation, offering an innovative therapeutic strategy for both acute management and prevention of POPF.

Notably, our approach differs fundamentally from the previously reported D‐peptide hydrogel strategy.^[^ ^3e^ ^]^ In the present work, the D‐Peptide mediated Microaggregate Degradation (DPMD) remains in a soluble state until it encounters pancreatic enzymes, whereupon it undergoes liquid–solid phase separation to form discrete microdroplets that eventually solidify into microaggregates. This transient microdroplet phase allows widespread dispersion and rapid enzyme capture, in contrast to a bulk hydrogel which is spatially constrained. Importantly, once enzymes are immobilized in the solid microaggregates, these tiny aggregates can be gradually cleared by the body's immune cells, as demonstrated in our study, potentially mitigating long‐term tissue accumulation. This mechanism offers a new insight into enzyme sequestration therapy by leveraging a dynamic phase‐separation process rather than a static gel, thereby providing improved distribution and a built‐in clearance pathway. Consequently, this research represents a significant leap forward in POPF treatment by introducing a novel method of enzyme regulation through LSPS while simultaneously contributing immensely to the broader field of LSPS by showcasing practical applications of peptide microaggregates in disease treatment. As such, it paves a path for future research endeavors and therapeutic innovations across various biomedical disciplines.

## 2 Results

### 2.1 The Design and Construction of DPMD

**Figure 1:**
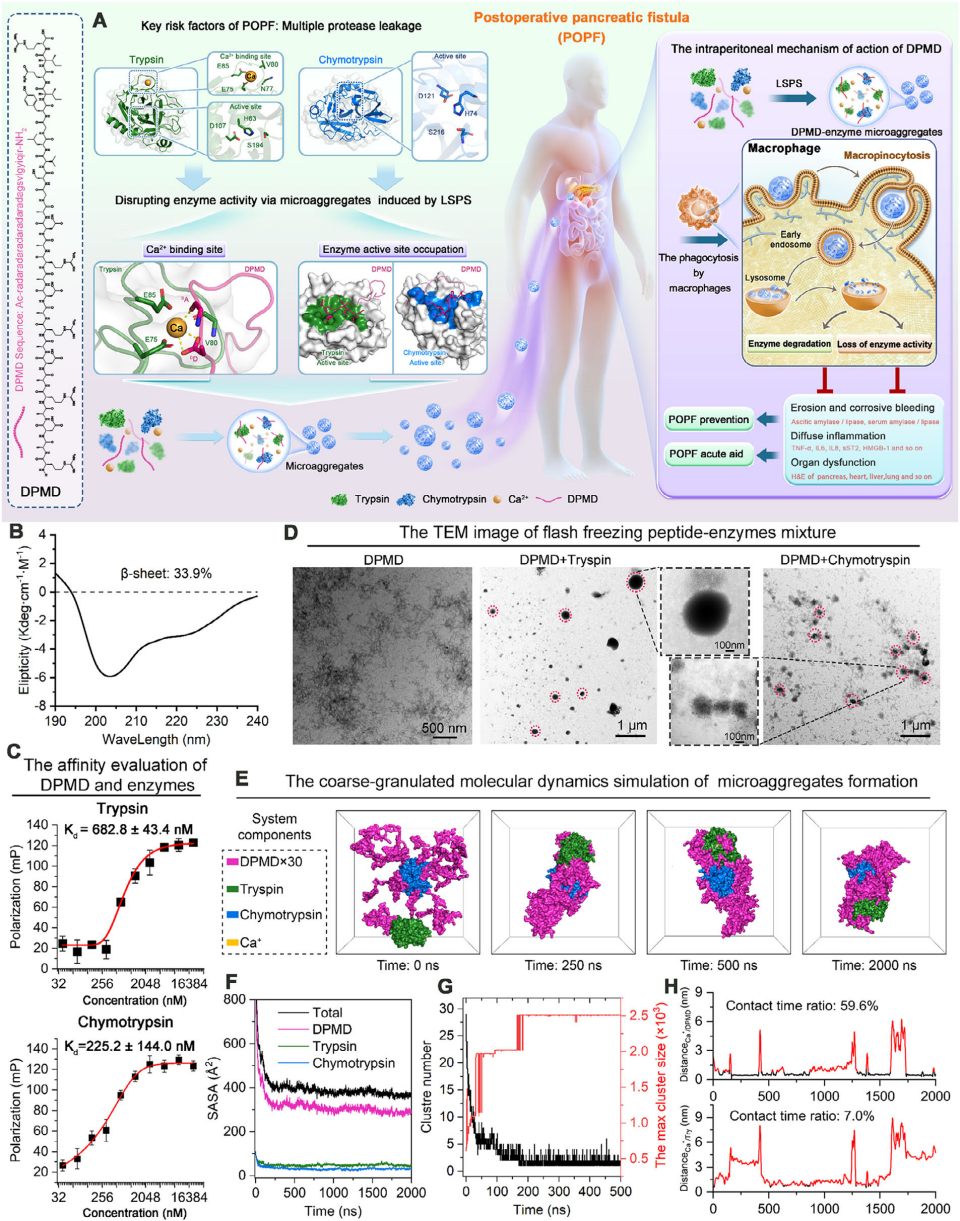
The Design and characterization of DPMD peptide for capturing digestive enzymes. A) Schematic illustration depicting the key risk factors, potential solutions, and underlying mechanisms associated with postoperative complications following pancreatic surgery. B) The CD spectrum of DPMD. C) The evaluation of binding affinity between DPMD peptide with trypsin or chymotrypsin (n = 3 biological replicates in each group, means ± SD). D) The TEM images of peptide‐protein microaggregates are induced by enzymes. E) The molecular dynamics simulation of coarse‐granulated peptide with enzymes. F) The SASA of different constituents and total. G) The number of clusters and the size of the largest cluster during the simulation. (H) The evaluation of Ca ion position changes during the simulation.

The DPMD is expected to exhibit an affinity for digestive enzymes in pancreatic fluid, orchestrating the assembly of intricate peptide‐enzyme microaggregates through LSPS (liquid–solid phase separation, Figure S1A, Supporting Information). In terms of digestive enzyme affinity, since pancreatic trypsin and chymotrypsin have been identified as the main culprits responsible for causing POPF by recognizing cationic and hydrophobic residues respectively to degrade peptides and proteins,^[^ ^6^ ^]^ it is imperative that the sequence of DPMD contains a sufficient quantity of cationic and hydrophobic residues. Furthermore, regarding the assembly of complex peptide‐enzyme microaggregates, previous studies have indicated that the relentless growth of fibrils plays a crucial role in LSPS transformation into anisotropic microaggregates through an entropy‐driven mechanism, which can be further enhanced by supramolecular polymerizations of synthetic components.^[^ ^7^ ^]^ Therefore, incorporating a peptide motif capable of self‐assembling into nanofibrils into DPMD would be highly advantageous. The classical nanofiber self‐assembly peptide (RADA)_4_, meeting the aforementioned requirements, emerges as a choice due to its unique suitability for enzyme sequestration. The (RADA)_4_ sequence is known to spontaneously self‐assemble into β‐sheet fibrils, forming nanostructured hydrogels that can trap biomolecules.^[^ ^3^, ^8^ ^]^ In the context of pancreatic enzymes, this sequence offers distinct advantages: i) Multiple Interaction Sites – The alternating positively and negatively charged residues present repetitive binding sites that can engage enzymes via electrostatic attraction and hydrogen bonding. For example, trypsin's substrate‐binding region contains negatively charged pockets that can interact with the arginine residues of DPMD, while chymotrypsin's hydrophobic pocket is flanked by regions that can form electrostatic contacts with DPMD's charged residues. ii) Protease Resistance – By utilizing D‐amino acids in DPMD, the peptide resists degradation by proteases, ensuring that it remains intact to perform its function in the protease‐rich postoperative environment. iii) Established Biocompatibility and Self‐Assembly – (RADA)_4_‐based peptides (including the D‐enantiomer) have been reported to be non‐cytotoxic and have been used in vivo as biomaterials, supporting their safety profile. In our case, DPMD retains the hallmark self‐assembly capacity: in the absence of target enzymes, it stays dissolved, but upon encountering trypsin or chymotrypsin rapidly undergoes phase transition into β‐sheet‐rich aggregates (as evidenced by circular dichroism in our supplementary data). We selected this sequence over other candidate self‐assembling peptides because (RADA)_4′_s specific combination of stability, self‐assembly propensity, and charge distribution is uniquely effective for sequestering serine proteases, as opposed to simply forming a generic hydrogel. By leveraging this design, we differentiate our approach from prior peptide assemblies and achieve targeted enzyme capture. Moreover, due to trypsin activation being reliant on calcium ions, a high concentration of Ca^2+^ ions was employed to expedite the formation of DPMD‐enzyme microaggregates by facilitating intermolecular bridging through insertion of the Ca^2+^ ion motif (D‐a.a seq: gsvlgyiqir) at the C‐terminal of DPMD (**Figure** **1**A). The DPMD design has now reached its culmination, poised to facilitate the uptake of anisotropic aqueous liquid microaggregates containing trypsin and chymotrypsin, thereby augmenting the propensity for macropinocytosis‐dependent internalization by macrophages (Figure 1A).

To validate the design, DPMD was synthesized using solid‐phase synthesis employing HOBt/HBTU and Fmoc chemistry, following a sequence schematic diagram depicted in Figure 1A. Subsequently, a single reverse column purification by High‐performance liquid chromatography (HPLC) yielded DPMD with purity exceeding 95% at a yield surpassing 60% (Figure S1B, Supporting Information). Circular dichroism (CD) spectra were recorded for a 40 µM DPMD sample in an aqueous solution containing 20% TFE at room temperature. The use of 20% TFE was necessary to obtain a clear spectrum, as it enhances peptide solubility and stabilizes secondary structure.^[^ ^9^ ^]^ The CD spectrum (Figure 1B) was analyzed by deconvolution, revealing ≈33.9% β‐sheet content, with the remaining ≈66% attributed to α‐helical and random coil structures (likely augmented by TFE's helix‐stabilizing properties). We did not perform CD on the irregular peptide‐enzyme aggregates because their large size and turbidity preclude reliable CD measurements. Furthermore, DPMD also showed a clear binding affinity toward Ca^2+^ ions (Figure S1C, Supporting Information). Notably, a control DPMD peptide lacking the Ca^2+^‐binding motif displayed a markedly reduced Ca^2+^‐binding capacity, highlighting the critical role of this motif in calcium coordination (Figure S1C, Supporting Information). Additionally, molecular docking predictions indicated that DPMD exhibits binding capabilities to both trypsin and chymotrypsin (Figure 1A; Figure S2, Supporting Information), which were further supported by fluorescence polarization experiments revealing high affinities between DPMD and Trypsin (682.8 nM) as well as Chymotrypsin (225.2 nM) (Figure 1C). In contrast, no appreciable binding was observed between DPMD and a non‐target protease, Proteinase K (Figure S3A, Supporting Information). Furthermore, when trypsin or chymotrypsin was pre‐incubated with the protease inhibitor aprotinin (rendering their active sites occupied), subsequent addition of DPMD resulted in only a minimal anisotropy change (Figure S3B,C, Supporting Information). This indicates that DPMD's binding to its target enzymes is largely inhibited when their active sites are blocked, suggesting that DPMD interacts with trypsin and chymotrypsin at or near those functional regions. To further confirm that DPMD's enzymatic targeting is specific and not a broad‐spectrum protein aggregation effect, we tested DPMD against other pancreatic enzymes and abundant plasma proteins. Fluorescently labeled DPMD was mixed with elastase, pancreatic lipase, α‐amylase, carboxypeptidase A, bovine serum albumin (BSA), and IgG, respectively, and fluorescence anisotropy was measured to detect binding. In stark contrast to the trypsin and chymotrypsin cases, DPMD showed no significant anisotropy increase with any of these non‐target proteins (Figure S3D–I, Supporting Information). This indicates that DPMD does not form stable complexes or aggregates with these proteins under the conditions tested. For example, anisotropy values for DPMD + elastase (or lipase, amylase, etc.) remained near the baseline (DPMD‐alone) level, whereas DPMD + trypsin produced a pronounced anisotropy elevation (reflecting peptide‐enzyme complex formation and microaggregate assembly). These results demonstrate the high specificity of DPMD: its phase‐separation and aggregating effect is effectively limited to trypsin and chymotrypsin, with negligible interaction with other enzymes or common proteins.

Moreover, the transmission electron microscope (TEM) image revealed a striking contrast between the anisotropic aqueous liquid microaggregates observed in the DPMD/Trypsin and DPMD/Chymotrypsin mixtures, and the nanofibrous structure present in DPMD alone (Figure 1D). This suggests that the binding of Trypsin and/or Chymotrypsin hinders the growth of DPMD into fibrils, restricting it to an intermediate state of microaggregates. To investigate the underlying mechanism of microaggregates formation, we employed molecular dynamics coarse‐grained simulation to simulate the self‐assembly process of LSPS‐induced peptide microaggregates. The simulation included 30 DPMD molecules, 1 trypsin molecule, 1 chymotrypsin molecule, and an unlimited number of water molecules containing Ca^2+^ ions in confined spaces. After energy minimization and phase equilibrium were achieved, a dynamic simulation was conducted for 2000 ns with the focus on hydrogen bond interactions that could lead to enthalpy changes. The results revealed that DPMD partially wrapped around the surfaces of the enzymes, leading to a rapid reduction in solvent‐accessible surface area (SASA) (Figure 1E,F, Supporting Information), as well as the formation of clusters (Figure 1G). These findings suggest that DPMD has a tendency to form peptide microaggregates with proteases through an entropy‐driven mechanism. Additionally, an interesting observation during the simulation was that positional analysis showed DPMD had contact with Ca^2+^ ions for ≈59.6% of the time compared to only 7% for trypsin (Figure 1H). This indicates that DPMD has a higher affinity for capturing Ca^2+^ ions than trypsin does, thereby facilitating an abundance of calcium ions available for accelerating microaggregates formation. Overall, these results demonstrate that DPMD efficiently recognizes trypsin and chymotrypsin and orchestrates their assembly into intricate peptide‐enzyme microaggregates through LSPS via an entropy‐driven mechanism.

### 2.2 The Generation of DPMD‐Trypsin/Chymotrypsin Microaggregates and their Subsequent Elimination by Macrophages

**Figure 2:**
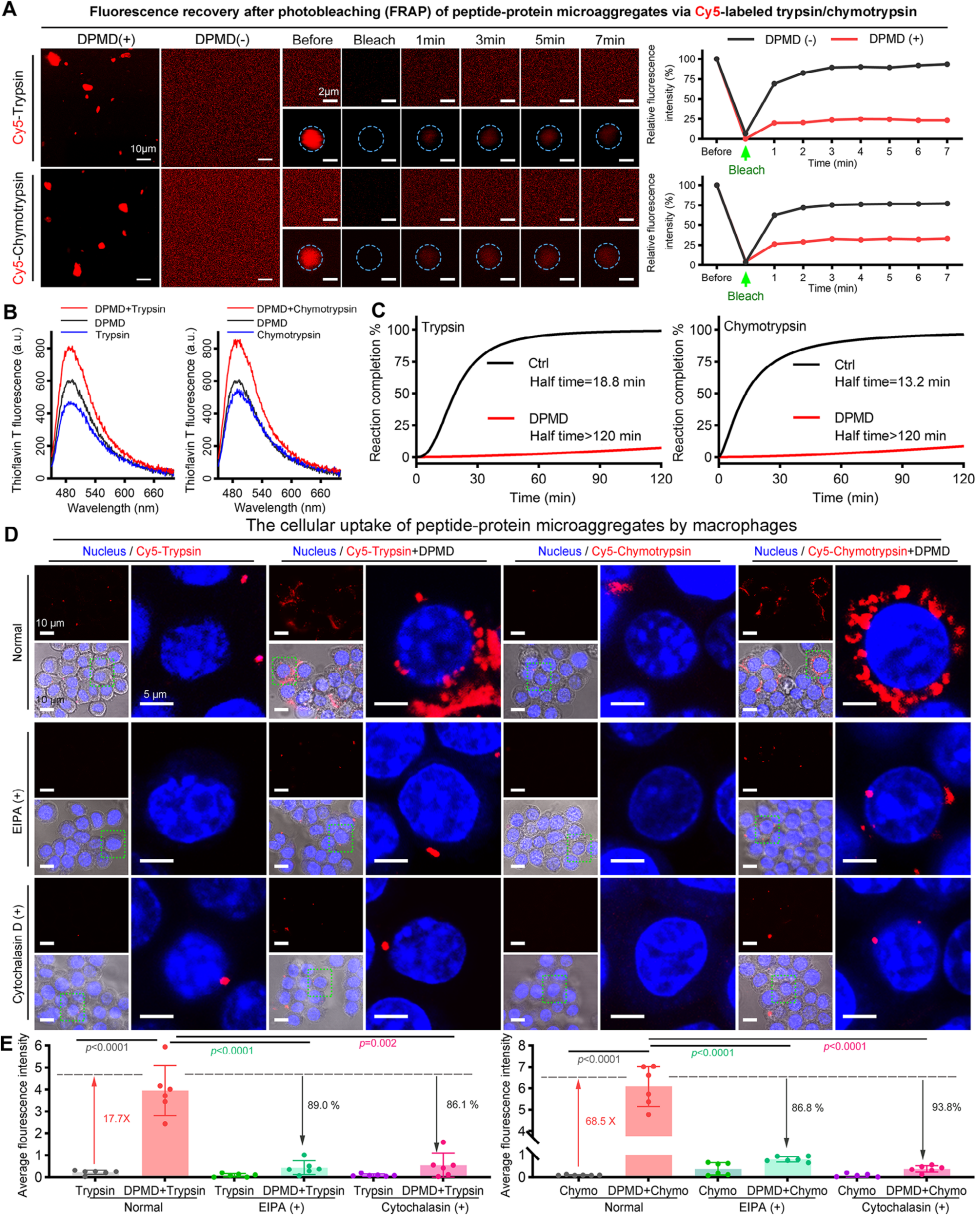
The generation of DPMD‐trypsin/chymotrypsin microaggregates and its subsequent elimination by macrophages. A) Representative time‐lapse FRAP images howling the pre‐bleach and recovery signals of DPMD‐treated trypsin^Cy5^ and chymotrypsin^Cy5^ in vitro. The trypsin^Cy5^ and chymotrypsin^Cy5^ without DPMD treatment was exhibited as control. Circles indicate the bleached area. Scale bars, 10 and 2 µm (zoomed‐in image). B) Fluorescence spectra of trypsin/chymotrypsin, DPMD, and trypsin/chymotrypsin with DPMD after ThT incubation. C) The inhibition evaluation of enzyme bioactivity for trypsin and chymotrypsin by DPMD. D) The cellular uptake of peptide‐protein microaggregates by macrophages, treating with/without EIPA or Cytochalasin D, as determined by Laser Scanning Confocal Microscopy (LSCM). E) The red fluorescence intensity of the cell area in different groups. The fluorescence values were calculated by Image J. The percentage labeled in the figure represents the degree of between‐group change compared to the DPMD‐treated enzyme group (n = 6 biological replicates in each group, means ± SD). The *p*‐value was calculated using an unpaired t‐test and labeled in the figures.

The addition of low concentrations of DPMD to Cy5‐labeled trypsin or chymotrypsin resulted in the formation of vibrant red irregular microaggregates, as observed through laser scanning confocal microscope (LSCM), which stood in stark contrast to the homogeneous red solution of trypsin or chymotrypsin without DPMD (**Figure** **2**A). Consistent with the microscopy images, a Dynamic Light Scattering analysis (Figure S4, Supporting Information) of DPMD, enzymes, and their mixtures revealed a broad size distribution of the peptide–enzyme complexes. The hydrodynamic diameters ranged from sub‐micron particles (≈0.2 µm) to multi‐micron aggregates (≈5 µm). This wide size range can be attributed to the aggregation mechanism: initial nanoscale assemblies of DPMD–enzyme can further cluster into larger microaggregates over time. Moreover, the LSPS‐driven peptide–enzyme aggregates do not readily fuse upon contact, indicating a solid or gel‐like consistency. These factors together result in heterogeneous aggregate sizes.

Next, these intriguing microaggregates were further investigated using fluorescence recovery after photobleaching (FRAP) experiments. In comparison to the DPMD‐free solution where fluorescence almost recovered within 4 min post‐bleaching, both DPMD‐trypsin and DPMD‐chymotrypsin microaggregates exhibited <25% fluorescence recovery even after 7 min (Figure 2A). To directly visualize the liquid–solid phase separation process, we performed time‐lapse confocal microscopy on mixtures of DPMD and trypsin/chymotrypsin. Initially, numerous small, spherical DPMD‐rich microdroplets appeared upon enzyme addition and gradually grew into larger droplets within minutes (Figure S5A, Supporting Information). Over time, these droplets matured into irregularly shaped solid aggregates, at which point fusion between aggregates did not occur. We next examined whether the enzyme–peptide aggregates remain stable under varying physiological conditions, such as changes in pH and enzyme concentration. Turbidity assays demonstrated that DPMD and trypsin/chymotrypsin mixtures exhibit robust phase separation, as indicated by increased turbidity, across a range of pH levels and enzyme concentrations (Figure S5B, Supporting Information). Importantly, when the protease activity was neutralized by pre‐incubation with the inhibitor aprotinin, no significant phase separation occurred –no enzyme aggregates were observed (Figure S5C, Supporting Information), underscoring that active enzyme‐peptide interactions drive the aggregation. Moreover, at pH 5.6 and pH 8.4, the mixtures still became cloudy and formed microscopically visible droplets/aggregates (Figure S5D, Supporting Information), albeit with slightly different turbidity magnitudes, indicating that moderate pH shifts do not prevent aggregate formation or induce aggregate dissolution once formed. Similarly, varying the enzyme‐to‐DPMD ratio showed that as long as DPMD was present in sufficient quantity to bind enzymes, stable aggregates formed and persisted (Figure S5E, Supporting Information).

Furthermore, to confirm the liquid nature of early‐stage droplets, we conducted fluorescence recovery after photobleaching (FRAP) on DPMD‐sequestered enzymes. In freshly formed DPMD–trypsin (or DPMD–chymotrypsin) droplets, bleached regions regained a portion of their fluorescence signal over several seconds (Figure S6A, Supporting Information), demonstrating partial molecular mobility and thus a dynamic liquid interior. In contrast, mature aggregates showed no fusion upon contact and no fluorescence recovery after photobleaching, consistent with a transition to a solid state (Figure S6B, Supporting Information). These observations provide direct evidence that the DPMD system undergoes true LSPS: it first behaves as a liquid droplet phase that is capable of internal rearrangement and droplet–droplet coalescence, then irreversibly solidifies to lock in the captured enzymes.

The Thioflavin T (ThT) staining further validated these findings, as it selectively labeled the degree of aggregation of peptide/protein microaggregates. As depicted in Figure 2B, the DPMD–trypsin and DPMD–chymotrypsin mixtures exhibit higher ThT fluorescence than either DPMD or enzyme alone, indicating the presence of more extensive peptide–enzyme aggregates when both components are combined. We attribute the increase in ThT signal to the formation of these DPMD–protease microaggregates, rather than to a trivial change in ionic strength. Notably, DPMD alone can also form β‐structured aggregates (yielding some ThT signal), but the significantly greater fluorescence in the mixtures suggests that co‐aggregation of DPMD with the enzymes produces additional β‐sheet‐rich structures. Meanwhile, the CD results demonstrate that there is no significant increase in the β‐sheet content of the enzyme alone or the DPMD‐enzyme mixtures (Figure S7, Supporting Information), suggesting that the observed ThT fluorescence in the DPMD‐enzyme complexes is due to the formation of more highly aggregated structures, rather than an increase in the quantity of β‐sheet structures. Notably, because the large microaggregates are turbid, CD spectra were collected under dilute conditions; consistent with Figure S7 (Supporting Information), no new β‐sheet content was detected in the DPMD‐enzyme mixtures compared to enzyme alone, reinforcing that the ThT increase reflects aggregate formation rather than additional β‐structure. More significantly, DPMD effectively suppressed the enzymatic activity of both Trypsin and Chymotrypsin to an almost complete extent (Figure 2C), implying that the formation of DPMD‐trypsin/chymotrypsin microaggregates can remarkably impede enzyme activity, presumably due to the physical isolation of enzymes from their substrates.

Due to the avidity of macrophages for engulfing protein/peptide aggregates via macropinocytosis pathway,^[^ ^10^ ^]^ another crucial feature of these LSPS‐derived microaggregates was their propensity to be eliminated by macrophages. To validate this, Cy5‐labeled Trypsin and Chymotrypsin, along with their DPMD mixtures, were co‐cultured with RAW264.7 macrophage cells. Flow cytometry analysis demonstrated that ≈80% of macrophages internalized DPMD–enzyme aggregates within 6 h, which represents a significantly higher uptake rate compared to the control groups (p < 0.01, Figure S8A, Supporting Information). Consistent with this observation, as shown in the first row of Figure 2D, both DPMD mixtures resulted in the uptake of red fluorescent aggregates by macrophages, whereas Trypsin and Chymotrypsin exhibited minimal internalization. Furthermore, pretreatment of macrophages with the macropinocytosis inhibitor EIPA or the actin polymerization inhibitor Cytochalasin D led to a substantial reduction in aggregate uptake (Figure 2D; Figure S8B, Supporting Information), suggesting that macropinocytosis is a predominant mechanism for internalization. Confocal imaging corroborated these findings, revealing markedly reduced intracellular fluorescence in cells treated with inhibitors (quantified in Figure 2E). Moreover, TEM imaging (Figure S9, Supporting Information) provided direct ultrastructural confirmation of macropinocytosis, visualizing large macropinosome‐like vesicles containing the DPMD–enzyme aggregates within macrophages.^[^ ^11^ ^]^ Collectively, these results indicate that DPMD can form microaggregates with Trypsin and Chymotrypsin, thereby enhancing their clearance by macrophages and potentially mitigating leakage‐induced damage during postoperative pancreatic fistula (POPF).

### 2.3 The DPMD Significantly Mitigated the Occurrence of POPF

**Figure 3:**
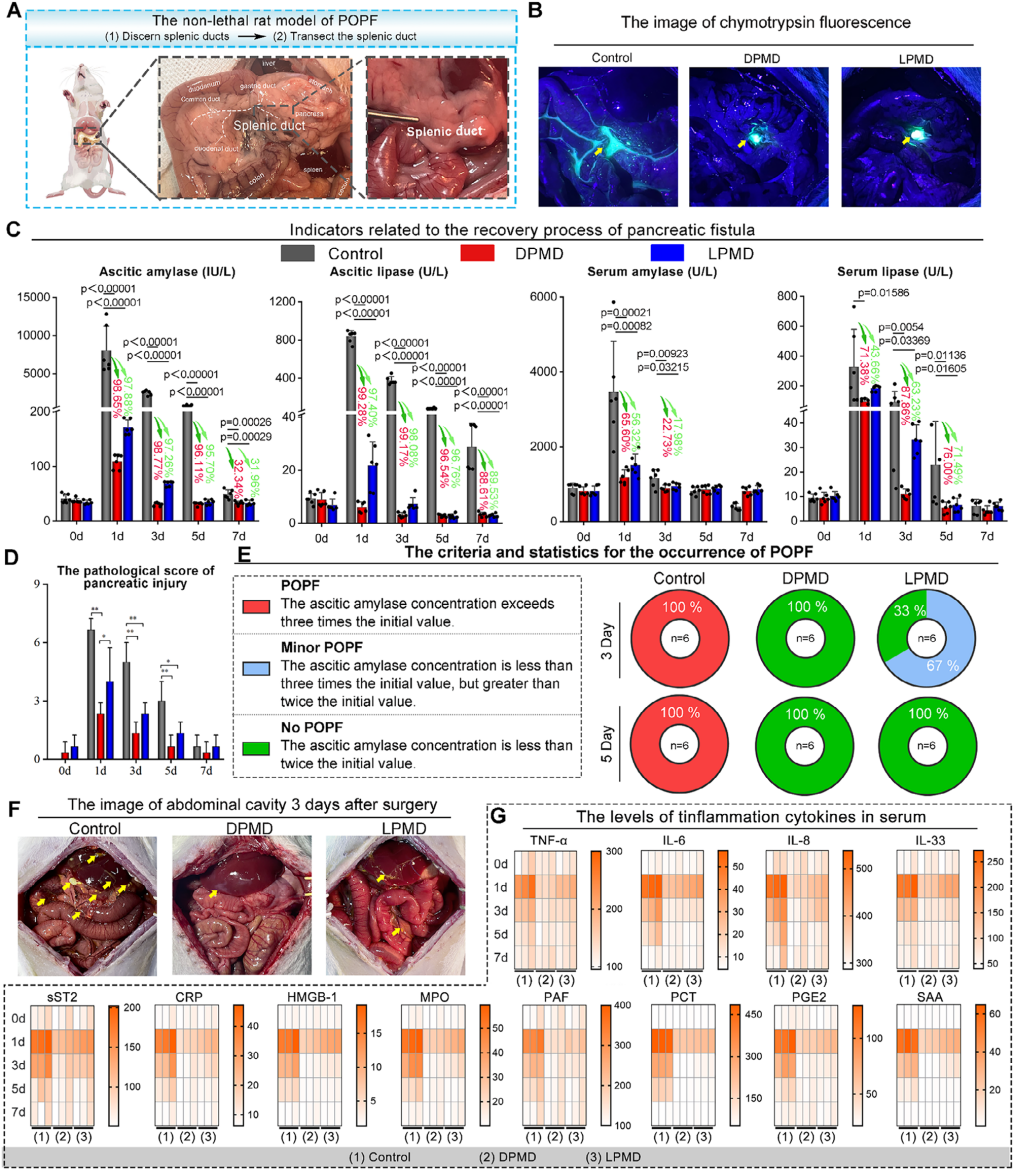
The evaluation of mitigating the occurrence of POPF. A) Schematic diagram of establishing a rat pancreatic splenic duct of non‐lethal transaction pancreatic fistula model. B) Imaging of chymotrypsin fluorescence in pancreatic splenic duct transection fistula including control, DPMD and LPMD groups. C) The pancreatic fistula recovery process related indicators: ascites amylase, ascites lipase, serum amylase, serum lipase. The dark/light green arrows and the labeled percentages in the figure represent the change of different groups. The *p*‐value was calculated using one‐way ANOVA and labeled in the figures. (n = 5 biological replicates in each group, means ± SD). D) The pathological score of pancreatic injury. (Data are presented as means ± SD, statistical analysis was performed using unpaired t‐test, * p < 0.05, ** p < 0.01, **p < 0.001). E) The criteria and statistics for the occurrence of POPF. F) Typical photo of exploratory laparotomy three days after surgery (yellow arrows indicate tissue lesion caused by pancreatic fistula). G) Heat maps of serum inflammatory factor levels in the control group, DPMD group, and LPMD group within 7 days after surgery.

To investigate the biofunction of DPMD in preventing POPF, a slowly occurring POPF model was established in rats by transecting the peripancreatic splenic duct to induce a continuous but low leakage of pancreatic juice (**Figure** **3**A). Following surgical modeling, DPMD, its L‐enantiomer LPMD, or normal saline (Ctrl) were topically applied on the incision site. After post operation, chymotrypsin leakage was assessed using a specific fluorescent dye. Notably, mock treatment resulted in significant leakage while DPMD exhibited nearly complete suppression of leakage with greater efficacy than LPMD (Figure 3B). By monitoring ascitic and serum amylase and lipase levels over a span of 7 days following surgical modeling (Figure 3C), we were able to make inferences about the pathological score (Figure 3D) and the occurrence of POPF (Figure 3E). The noteworthy observation is that all the mock‐treated rats experienced POPF on day 3 following surgical modeling, and this phenomenon persisted until day 5 (Figure 3E). In contrast, LPMD reduced both the occurrence and severity of POPF, resulting in 33% of rats being healthy and only 67% experiencing minor POPF on day 3; furthermore, all rats fully recovered by day 5 (Figure 3E). Most significantly, DPMD significantly suppressed the incidence of POPF, thereby further reinforcing the conclusion that DPMD demonstrated superior efficacy in preventing leakage compared to LPMD. Additionally, H&E staining of pancreatic tissue revealed that DPMD treatment led to reduced edema and inflammatory cell infiltration (Figure S10, Supporting Information), resulting in minimal abdominal damage compared to mock and LPMD‐treated tissues (Figure 3F). Furthermore, both DPMD and LPMD treatments decreased inflammation markers including TNF‐α, IL‐6, IL‐8, IL‐33, sST2, CRP, HMGB1, MPO, PAF, PCT, PGE2, and SAA in rats’ serum (Figure 3G). These results demonstrate that DPDM effectively reduces the incidence of POPF.

### 2.4 The Utilization of DPMD Proves to be an Efficacious Acute Intervention for the Management of Lethal POPF

**Figure 4:**
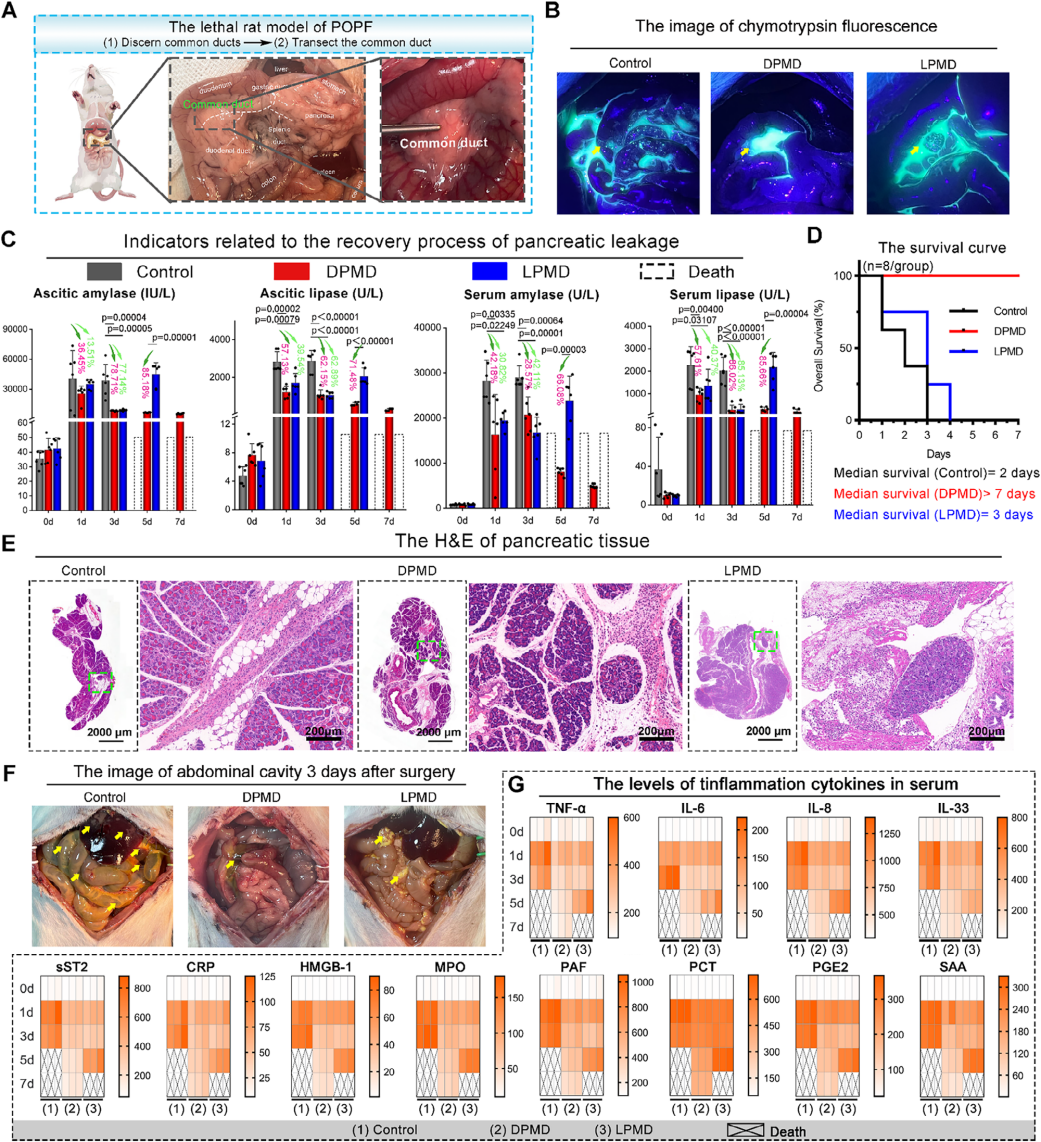
The evaluation of the management of lethal POPF. A) Schematic diagram of establishing a rat pancreatic common duct of lethal transaction pancreatic fistula model. B) Imaging of chymotrypsin fluorescence three day after surgery including control, DPMD and LPMD groups. C) The pancreatic fistula recovery process related indicators: ascites amylase, ascites lipase, serum amylase, serum lipase. The red/green percentage labeled in the figure represents the degree of between‐group change. The *p*‐value was calculated using one‐way ANOVA and labeled in the figures. Survival curves of pancreatic common duct transection fistula models treated with control, DPMD and LPMD. D) The survival rates of rats treating DPMD or LPMD. E) Typical H&E staining photograph of the pancreas three days after surgery. F) Typical photo of exploratory laparotomy three day after surgery (yellow arrows indicate tissue lesion caused by pancreatic fistula). G) Heat maps of serum inflammatory factor levels in the control group, DPMD group, and LPMD group within 7 days after surgery.

In order to challenge the efficacy of DPMD in emergency treatments for POPF, a lethal rat model of POPF was established through transection of the main pancreatic duct. After 8 h post‐surgical modeling, evidence of severe POPF was observed through the presence of leaky Chymotrypsin (Figure S11, Supporting Information). At this critical juncture, DPMD, LPMD or an equal volume normal saline solution were administered via syringe injection into the abdominal cavity near the pancreas. Subsequently, after 3 day and following an opening laparotomy and staining with Chymotrypsin, it became evident that DPMD effectively halted the leakage almost entirely – a stark contrast to the exaggerated diffuse leakage observed (**Figure** **4**B). Moreover, in comparison to the mock treatment, DPMD exhibited a statistically significant reduction in ascitic and serum amylase and lipase levels after surgical modeling (Figure 4C), leading to a decrease in the pathological score of POPF (Figure S12, Supporting Information). It is worth noting that in this lethal POPF model, DPMD demonstrated greater potency than LPMD (Figure 4B,C; Figure S12, Supporting Information), possibly due to its proteinase resistance attribute resulting from D‐peptide engineering. Consequently, all eight rats subjected to the mock treatment succumbed to three days post‐surgical modeling, while LPMD only extended the median survival from 2 days in Ctrl to 3 days in LPMD (Figure 4D). Excitingly, none of the rats treated with DPMD died during the entire seven‐day observation period following surgical modeling (Figure 4D), thus highlighting the exceptional efficacy of DPMD. Additionally, H&E staining of pancreatic tissue revealed that DPMD treatment led to reduced edema and inflammatory cell infiltration (Figure 4E), resulting in minimal abdominal damage compared to tissues treated with mock or LPMD (Figure 4F). Furthermore, both DPMD and LPMD treatments decreased inflammation markers including TNF‐α, IL‐6, IL‐8, IL‐33 sST2, CRP, HMGB1, MPO, PAF, PCT, PGE2 and SAA present in rat serum (Figure 4G). These findings demonstrate that utilizing DPMD proves highly effective as an acute intervention for managing potentially lethal POPF.

Furthermore, in ex vivo experiments using ascitic fluid harvested from this lethal POPF model, DPMD was found to trigger the formation of micron‐scale peptide–enzyme aggregations in the fluid (Figure S13, Supporting Information). These microaggregates remained as distinct mircoaggregates that did not fuse with each other, demonstrating stable phase‐separated structures even in the complex ascitic environment. FRAP analysis of individual mircoaggregates revealed minimal fluorescence recovery after photobleaching, confirming that the sequestered enzymes are localized within solid aggregates. Consistent with our in vitro observations, the proteolytic activity of trypsin and chymotrypsin was markedly suppressed when the enzymes were captured within DPMD‐induced microaggregates in ascitic fluid. This ex vivo evidence confirms that DPMD mitigates POPF via an LSPS‐based mechanism: by assembling leaking pancreatic enzymes into non‐fusing microaggregates, DPMD effectively isolates and inactivates the enzymes, thereby preventing proteolytic damage to surrounding tissues.

### 2.5 The DPMD Exhibits a Favorable Biosafety and Biocompatibility Profile

For further evaluation of the clinical translational potential of DPMD, we next evaluated the biocompatibility of DPMD in vitro and in vivo. Fibroblast (NIH‐3T3) and endothelial cell (HUVEC) cultures exposed to DPMD remained highly viable (>90% viability by CCK8 assay) with no significant difference from untreated controls (Figure S14A, Supporting Information). Even when DPMD was pre‐incubated with trypsin or chymotrypsin to form enzyme–peptide aggregates, cell viability was unaffected, and flow cytometry for apoptotic markers (Annexin V/PI) showed no increase in apoptosis or necrosis due to treatment (Figure S14B, Supporting Information). These results indicate that DPMD and the sequestered‐enzyme aggregates are not cytotoxic to mammalian cells in culture.

To assess the fate of DPMD in vivo, rats were administered DPMD intraperitoneally and monitored using in vivo fluorescence imaging. The DPMD peptide (labeled with a fluorescent tag) showed a broad initial distribution within the peritoneal cavity and moderate uptake in filtering organs (liver, kidneys, spleen). Encouragingly, the fluorescence intensity in these organs diminished over time, becoming barely detectable by day 6 post‐injection (Figure S14C, Supporting Information). In parallel, fluorescence appeared in urine samples (Figure S14D, Supporting Information), indicating renal excretion of DPMD or its metabolites. These data suggest that DPMD does not accumulate persistently in tissues; instead, it undergoes clearance.

**Figure 5:**
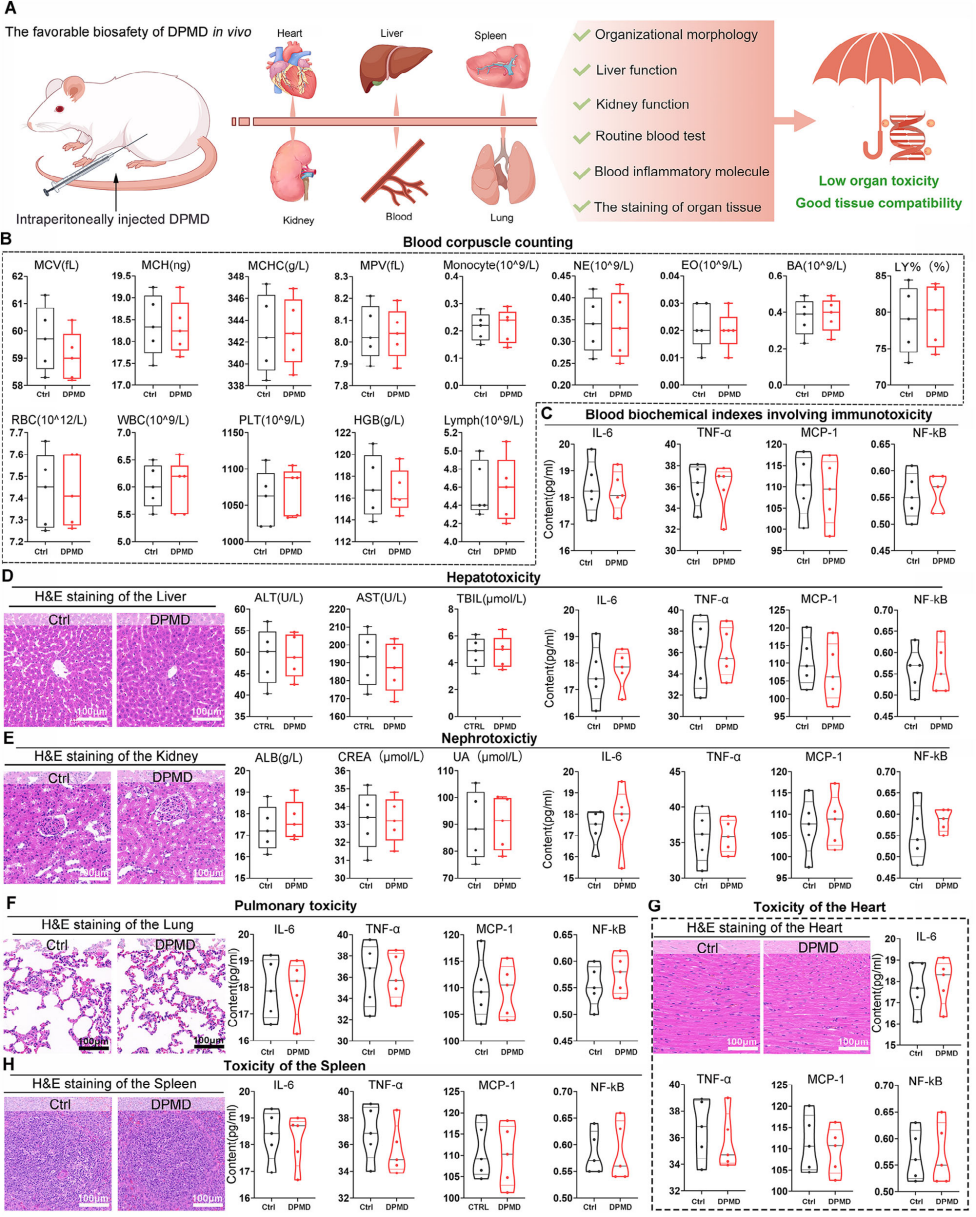
The favorable biosafety and biocompatibility of DPMD. A) The schematic diagram of therapeutic safety benefited from the DPMD. B&C) The blood corpuscle counting and immunotoxicity‐related blood biochemical indexes of rats treated with the DPMD or PBS using intraperitoneal administration. D) Hepatotoxicity measured by pathological section of liver, ALT (glutamic‐pyruvic transaminase), AST (glutamic‐oxalacetic transaminase), total bilirubin (TBIL) and inflammatory cytokines (IL‐6, TNF‐α, MCP‐1and NF‐κB) of liver. E) Nephrotoxicity test, including pathological section of kidney, CR (creatinine), BUN (Blood Urea Nitrogen), albumin (ALB) and inflammatory cytokines (IL‐6, TNF‐α, MCP‐1and NF‐κB) of kidney. F) Toxicity evaluation of lung displayed by H&E staining images of lung sections and IL‐6, TNF‐α, MCP‐1and NF‐κB cytokines content in alveolar lavage fluid. G) Cardiotoxicity reflected by pathological section and inflammatory cytokines (IL‐6, TNF‐α, MCP‐1and NF‐κB) infiltrating in heart. H) The H&E staining of spleen sections, and IL‐6, TNF‐α, MCP‐1and NF‐κB cytokines were involved in safety detection of DPMD or PBS in spleen. The data from each group were presented as the mean ± SD. Scale bar: 100 µm. Statistical analysis in B‐G was performed using unpaired t‐test.

For in vivo safety, rats receiving an intraperitoneal injection of DPMD were monitored for systemic toxicity and immune response. Clinical chemistry and hematological analyses at 7 days post‐treatment showed no aberrations: blood cell counts (red and white cells, platelets) remained within normal limits, and pro‐inflammatory cytokine levels (IL‐6, TNF‐α, MCP‐1, NF‐κB) in serum were indistinguishable from saline‐treated controls (**Figure** **5**B,C). Liver function tests (ALT, AST, total bilirubin) and kidney function markers (creatinine, BUN, albumin) showed no signs of organ damage or dysfunction attributable to DPMD (Figure 5D,E). Consistently, histopathological examination revealed no microscopic evidence of toxicity in major organs. Liver sections displayed normal hepatocyte morphology with no inflammation (Figure 5D), kidney glomeruli and tubules were intact (Figure 5E), and lung tissue showed no edema or inflammatory cell infiltrates (Figure 5F). The heart and spleen likewise appeared normal, with no myocardial inflammation or splenic white pulp enlargement (Figure 5G,H). Taken together, these findings demonstrate that DPMD treatment was well‐tolerated in vivo, with no detectable short‐term toxicity or immune adverse effects in our models. We acknowledge that longer‐term studies will be needed, but these initial results support the biosafety of DPMD as a therapeutic agent.

## 3 Discussion

The crux of POPF lies in the exudation of vital digestive enzymes, specifically Trypsin and Chymotrypsin, which initiate a process of “self‐digestion” within the muscular tissue present in the abdominal cavity.^[^ ^3c^ ^]^ As a result, this self‐digestive phenomenon not only compromises the integrity of the anastomosis but also expedites the leakage of pancreatic juice containing these potent digestive enzymes.^[^ ^3^, ^12^ ^]^ Therefore, interrupting this vicious cycle is of utmost importance in both the treatment and prevention of POPF. Currently, conventional methods for preventing and treating postoperative pancreatic fistula (POPF) primarily rely on small‐molecule protease inhibitors or sealing materials. While these approaches can help manage symptoms, they have notable limitations. Enzyme inhibitors, such as gabexate mesylate, can reduce protease activity, but they often fail to provide long‐term inhibition and may cause systemic side effects, such as thrombocytopenia and liver dysfunction.^[^ ^13^ ^]^ Sealing materials, including fibrin glues, can prevent pancreatic fluid leakage but do not directly address the enzymatic damage caused by digestive enzymes like trypsin and chymotrypsin, which continue to degrade surrounding tissue.^[^ ^14^ ^]^

Herein, DPMD, a D‐peptide, has been developed to exhibit an affinity for trypsin and chymotrypsin while coordinating the assembly of intricate peptide‐enzyme microaggregates via LSPS. This sequestration effectively isolates the enzymes from their substrates, proficiently inhibiting their activity and preventing further damage to pancreatic tissue. Notably, our supplemental specificity studies did not reveal binding of DPMD to non‐target enzymes like elastase or to abundant proteins like albumin, indicating a low risk of sequestering proteins unrelated to POPF pathology. This specificity is crucial, as it means DPMD is unlikely to mop up beneficial proteins or enzymes indiscriminately, thereby limiting potential side effects. Furthermore, these peptide‐enzyme microaggregates are efficiently taken up by macrophages through macropinocytosis, leading to their clearance by macrophages in the inflamed area. The current experimental design mainly relied on macropinocytosis inhibitors and could not fully rule out other non‐traditional pathways;^[^ ^15^ ^]^ future experiments with more refined methods are necessary to clarify the precise mechanisms of microaggregate uptake by macrophages.

Compared to traditional therapies, DPMD not only halts enzymatic activity but also facilitates the efficient removal of enzymes from sites of inflammation, offering an innovative therapeutic strategy for both acute management and safeguarding against POPF. The utilization of LSPS to control enzyme activity in vivo represents significant progress in the broader domain of LSPS applications. Previous investigations have mainly concentrated on employing LSPS to regulate intracellular biomolecular processes, such as stress granule formation and protein localization.^[^ ^2^, ^16^ ^]^ Our research extends these studies by demonstrating the capacity of LSPS to sequester harmful enzymes in an extracellular context, providing a potent new means for the treatment of enzyme‐related diseases like POPF.

In this study, we refer to liquid–solid phase separation (LSPS) as a two‐step process in which initially a liquid‐liquid phase separation occurs, producing enzyme–peptide‐rich liquid microdroplets, followed by a maturation into solid‐like aggregates. This is distinct from mere aggregation or precipitation. The hallmark features of a phase‐separated liquid droplet – such as the ability to fuse with one another and exchange contents – were observed in our system at early time points (e.g., DPMD droplets coalescing and partial FRAP recovery; see Results). Such behavior confirms a true phase‐separation event rather than nonspecific aggregation. We note that once the droplets solidify, they no longer fuse (non‐reversibility), which is an intended outcome to stably immobilize the enzymes. Thus, while classical phase separation is often fully reversible, our LSPS mechanism involves an irreversible solidification step. By clearly defining LSPS in this manner, we aim to use the term in a scientifically rigorous way, distinguishing our observations from conventional aggregation phenomena.

Our DPMD‐based therapy is envisioned for local administration in the context of abdominal surgery. A key advantage is that DPMD can be delivered as a simple aqueous solution (e.g., via a fine catheter or syringe) into the postoperative site, forming microdroplets in situ only upon contact with enzyme‐rich fluids. This triggerable assembly ensures that catheters are unlikely to clog during delivery, as the peptide remains monomeric until it mixes with pancreatic enzymes at the target location. The process is amenable to intraoperative use (spraying or dripping into the surgical area) or postoperative use through drainage catheters. In terms of production, DPMD is a 16‐residue peptide synthesized using standard solid‐phase peptide synthesis; thus, scalability to clinical‐grade manufacturing is feasible, similar to other peptide drugs already in use. Potential challenges were carefully considered: for example, the accumulation of peptide in a drain. We observed a progressive decline in peptide signal in vivo (Figure S14C,D, Supporting Information), suggesting that renal excretion contribute to eliminating the DPMD. This natural clearance route mitigates concerns about long‐term residue of the material. We acknowledge that further studies in large animal models and eventually humans will be necessary to confirm these aspects, including optimal delivery methods, dosing, and any unforeseen complications (such as ensuring the peptide does not impede normal healing). Nonetheless, these initial considerations highlight a promising translational pathway for DPMD therapy, with practical deliverability and manageable safety profile.

While DPMD demonstrated superior efficacy in preventing pancreatic fluid leakage compared to its L‐peptide counterpart (LPMD) in our animal models, we acknowledge that this approach is not a panacea and comes with important considerations. The use of a D‐amino acid peptide, although conferring resistance to proteolysis, raises questions about long‐term fate and immunogenicity. D‐peptides are generally less recognizable by the immune system; however, we cannot rule out immune reactions. Extended exposure could potentially lead to antibody formation against DPMD or the enzyme–DPMD aggregates. Our current studies, limited to acute and subacute time frames, did not show significant toxicity or inflammation, but long‐term biocompatibility remains to be evaluated. Additionally, the regulatory pathway for a D‐peptide therapeutic will require careful attention: manufacturing must ensure the peptide is of high purity and racemization‐free, and comprehensive toxicology studies (including chronic toxicity and reproductive toxicity) will be needed. We have addressed biosafety in this revision (showing no overt toxicity in vitro or in vivo over 7 days), yet we emphasize that these findings are preliminary. Finally, although our results in a rat POPF model are encouraging, clinical efficacy in humans is not guaranteed; factors such as human immune responses, variability in enzyme levels, and differences in postoperative management could influence outcomes. Therefore, we present DPMD as a promising and potentially effective strategy that substantially improves upon current treatments in preclinical trials, while recognizing that further research and cautious evaluation are required before claiming it as a definitive solution.

In conclusion, this research represents a remarkable leap forward in the treatment of POPF by introducing an innovative method of enzyme regulation through liquid–solid phase separation (LSPS), while simultaneously making immense contributions to the broader field of LSPS by showcasing practical applications of peptide microaggregates in disease treatment. As such, it paves a path for future research endeavors and therapeutic innovations across various biomedical disciplines.

## Conflict of Interest

The authors declare no conflict of interest.

## Supporting information



Supporting Information

## Data Availability

The data that support the findings of this study are available from the corresponding author upon reasonable request.
