# Supplementary material for: Regulating Enzyme Activity via Microaggregates Mediated by Phase Separation
Source: Adv Sci (Weinh). 2025 Jul 23;12(38):e09209. doi: 10.1002/advs.202509209 (PMC12520456; doi:10.1002/advs.202509209)
Supplement: Supplementary file 1 — Supporting Information [file ADVS-12-e09209-s001.pdf]

ADVANCED SCIENCE
Open Access

Supporting Information

for *Adv. Sci.*, DOI 10.1002/advs.202509209

Regulating Enzyme Activity via Microaggregates Mediated by Phase Separation

*Yang Wang, Juzheng Yuan, Niu Dai, Yanlin Ji, Wenguang Yang, Xiao Li, Siqi Yan and Jin Yan**

Supplementary Materials

Regulating Enzyme Activity *via* Microaggregates Mediated by Phase Separation

Yang Wang[1,2,3,†], Juzheng Yuan[3,4†], Niu Dai[3,4], Yanlin Ji[1,3], Wenguang Yang[5], Xiao Li[3,4], Siqi Yan[1,2], and Jin Yan[1,2*]

[1] Department of Hepatology, The Second Affiliated Hospital of Xi'an Jiaotong University, Xi'an, 710004, PR. China.

[2] Department of Tumor and Immunology in precision medical institute, Western China Science and Technology Innovation Port, The Second Affiliated Hospital of Xi'an Jiaotong University, Xi'an, 710004, China.

[3] Department of Hepatobiliary Surgery, Xijing Hospital, The Fourth Military Medical University, Xi'an 710032, China

[4] Department of General Surgery, Xijing Hospital, The Fourth Military Medical University, Xi'an 710032, China

[5] Department of Medical Oncology and Department of Talent Highland, The First Affiliated Hospital of Xi'an Jiaotong University, Xi'an 710061, PR. China.

[†] These authors contributed equally.

[*] Corresponding authors:
Email: yanjin19920602@xjtu.edu.cn (J. Yan).

1. Supplemental Table

Chemicals list	
Chemical Name	Brand
$CaCl_2$	Aladdin
Calcium Assay Kit	Beyotime
Amiloride	MCE
Cytochalasin D	Merck
Trypsin	Diamond
Chymotrypsin	Aladdin
Elastase	Sangon
Lipase	Macklin
Amylase	Aladdin
Carboxypeptidase	Yingxinbio
Albumin	Psaitong Biotechnology
Immunoglobulins	Solarbio
Aprotinin (trypsin/chymotrypsin inhibitor)	Yeasen Biotechnology
Trypsin activity assay kit	Solarbio
Chymotrypsin activity assay kit	Solarbio
DMEM culture medium	Gbico
DAPI	Beyotime
ThT	Aladdin
Hematoxylin and Eosin Staining Kit	Beyotime
MeO-Succ-Arg-Pro-Tyr-AMO solution	AAT Bioquest
Chemicals used in this study without specified.	Sigma-Aldrich

Table 1. The chemicals list of this study

2. Supplemental Figures

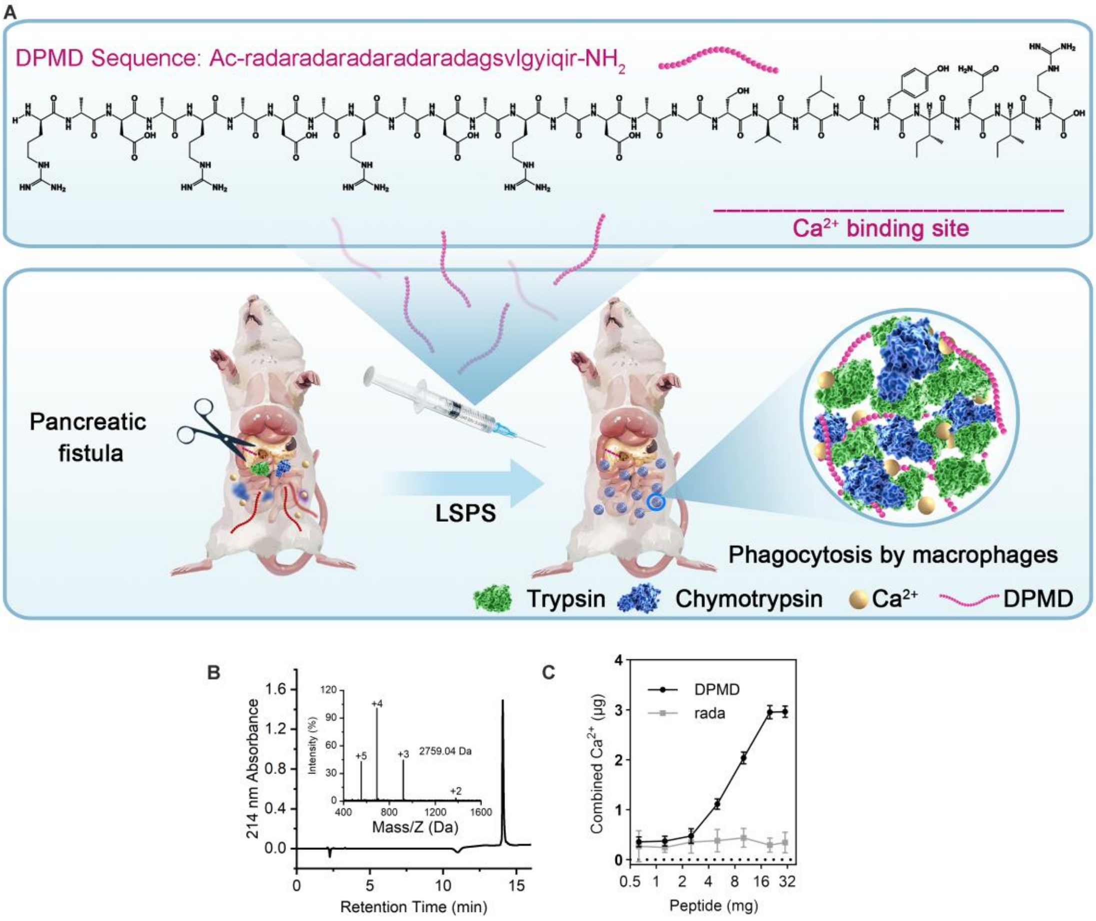

Figure S1. (**A**) The schematic diagram of the design and construction of DPMD. (**B**) The characterization of synthesized Ac-radaradaradaradaradagsvlgyiqir-NH_2 (DPMD) by LC-MASS and HPLC. (**C**) The characterization of the ability of DPMD and rada (DPMD control: the Ca^{2+} binding motif was removed) to bind Ca^{2+}.

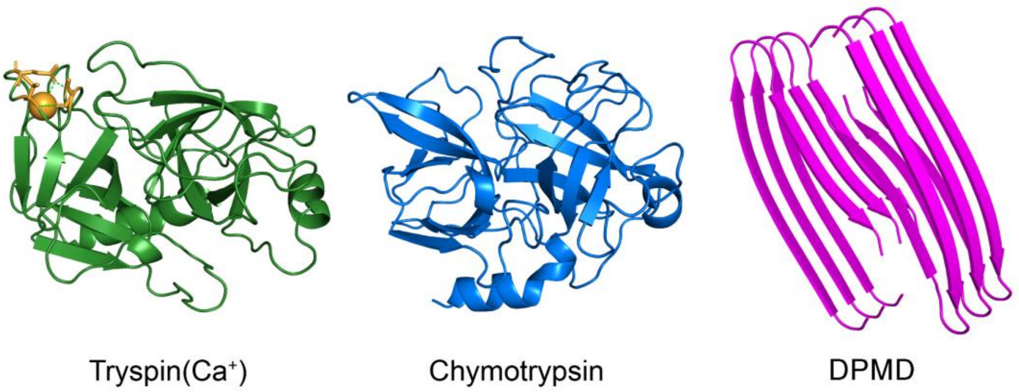

Figure S2. Schematic diagram of the structure of the Trypsin, Chymotrypsin, and DPMD.

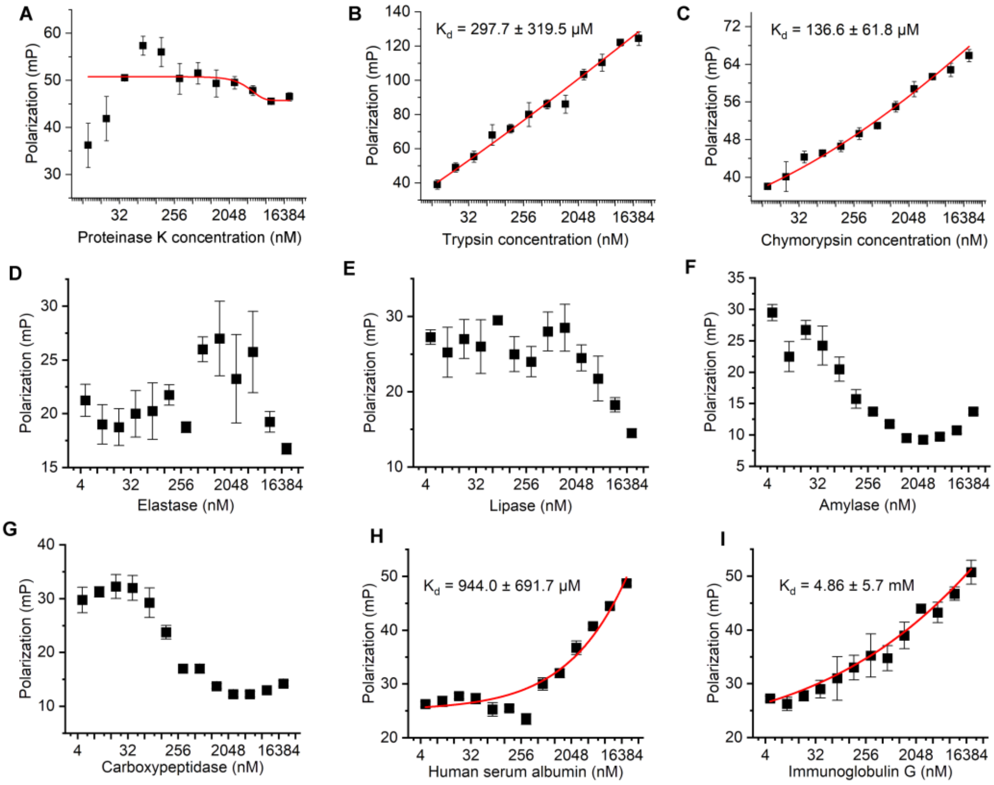

Figure S3. The fluorescent anisotropy evaluation of binding affinity between DPMD peptide with Proteinase K (**A**), and inhibitor-saturated and blocked trypsin (**B**) and chymotrypsin (**C**). Trypsin/Chymotrypsin was saturated and blocked by aprotinin (Yeasen Biotechnology, trypsin/chymotrypsin inhibitor). (**D-H**) The fluorescent anisotropy evaluation of binding affinity between DPMD peptide with elastase (**D**), lipase (**E**), amylase(**F**), carboxypeptidase (**G**), albumin (**H**) and immunoglobulins (**I**).

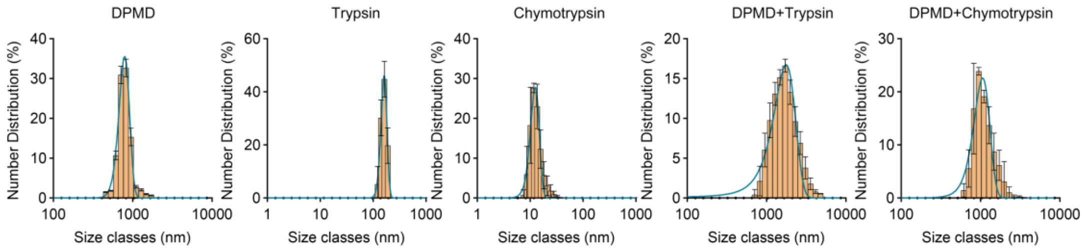

Figure S4. The DLS analysis of trypsin, chymotrypsin and DPMD with/without trypsin or chymotrypsin.

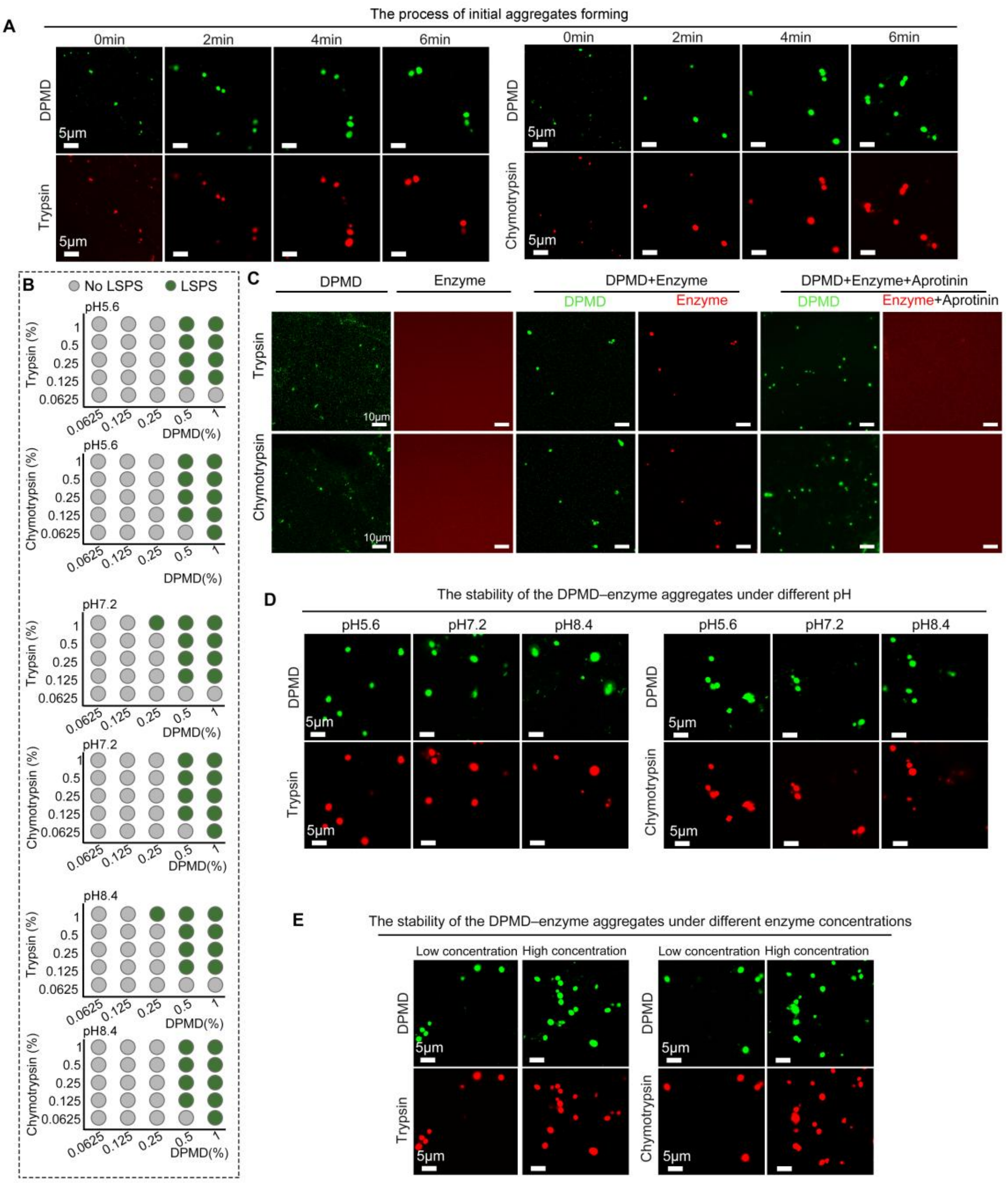

Figure S5. (**A**) The CLSM images of DPMD with enzyme and DPMD without enzyme under different time. (**B**) Turbidity measurements result illustrating the phase separation of DPMD-treated trypsin and chymotrypsin at different concentrations or pH shifts. % represents the mass fraction of the DPMD/trypsin/chymotrypsin. (**C**) The CLSM images of DPMD with enzyme, DPMD without enzyme and DPMD with enzyme and aprotinin. (**D&E**) The CLSM images of DPMD with enzyme and DPMD without enzyme under different pH (**D**) or enzyme concentrations (**E**).

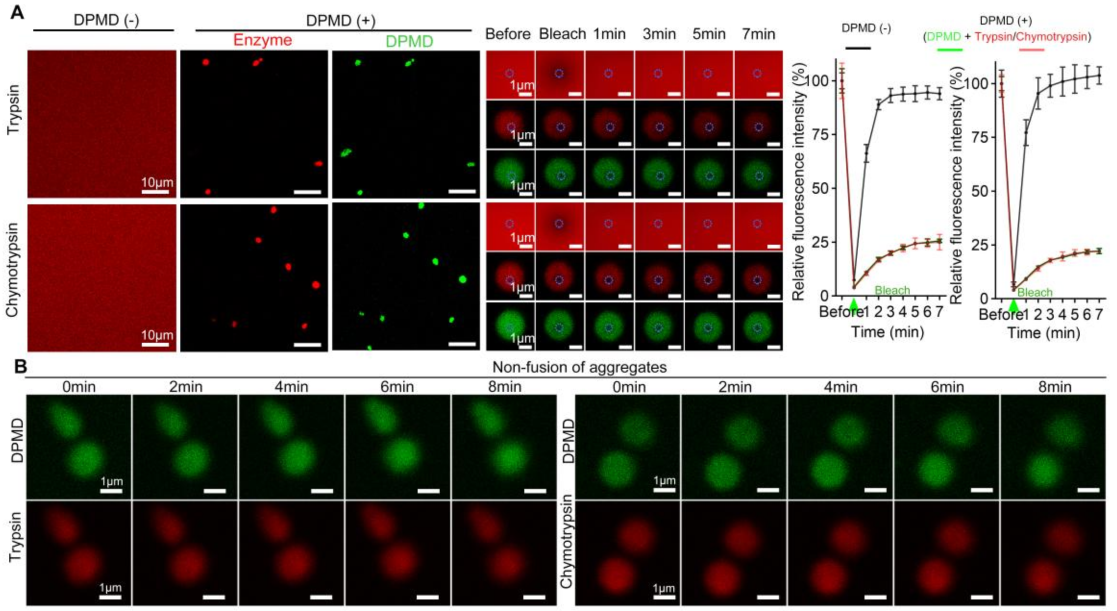

Figure S6. (**A**) Representative time-lapse FRAP images howling the pre-bleach and recovery signals of DPMD-treated trypsin^{Cy5} and chymotrypsin^{Cy5} *in vitro*. The trypsin^{Cy5} and chymotrypsin^{Cy5} without DPMD treatment was exhibited as control. Circles indicate the bleached area. The scale bars indicate a length of 10 μm and 1μm (Zoomed-in image). The fluorescence intensity was determined by averaging the results from five independent aggregate (n=5). (**B**) Time-lapse images of DPMD-trypsin/chymotrypsin mixture aggregates, illustrating the non-fusion of two aggregates.

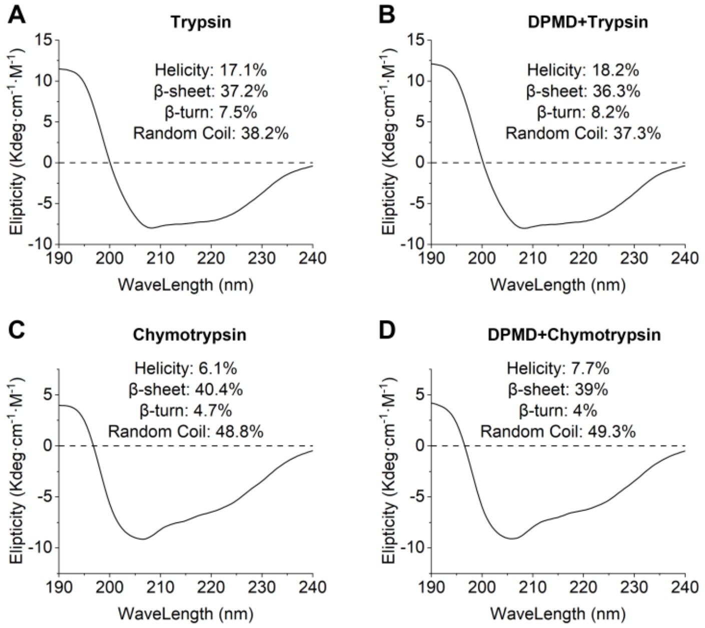

Figure S7. (**A&B**) Circular dichroism (CD) spectra of trypsin in the presence (**A**) and absence (**B**) of DPMD; (**C&D**)

CD spectra of chymotrypsin in the presence (**C**) and absence (**D**) of DPMD. The signal of the peptide DPMD was subtracted in the protease group containing it.

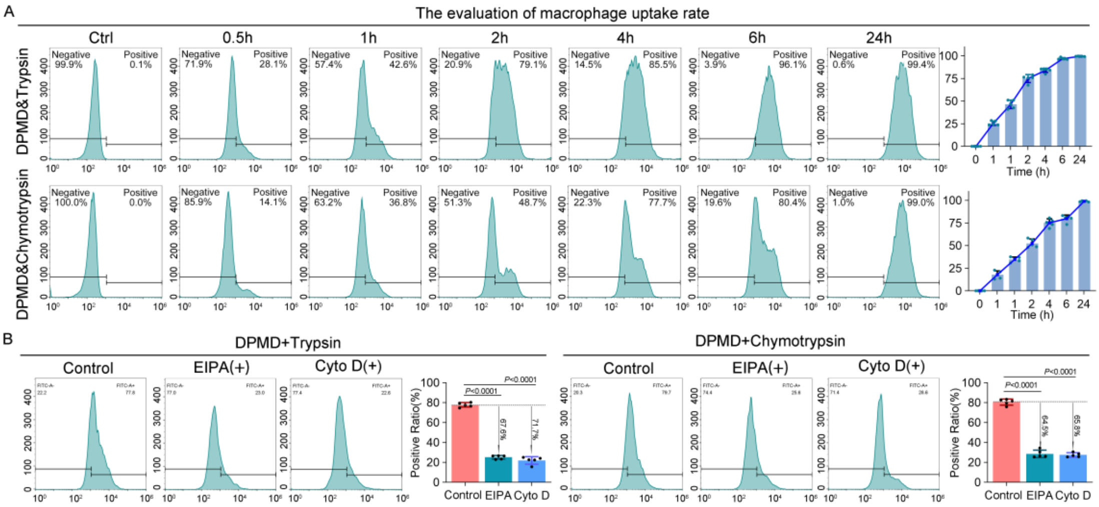

Figure S8. (**A**) The cellular uptake of peptide-protein microaggregates by macrophages as determined by flow cytometry. (**B**) The cellular uptake of peptide-protein microaggregates by macrophages, treating with/without EIPA or Cytochalasin D, as determined by flow cytometry. The fluorescence values were calculated by Image J. The percentage labeled in the figure represents the degree of between-group change compared to the DPMD-treated enzyme group. The *p*-value was calculated using a two-tail t-test and labeled in the figures.

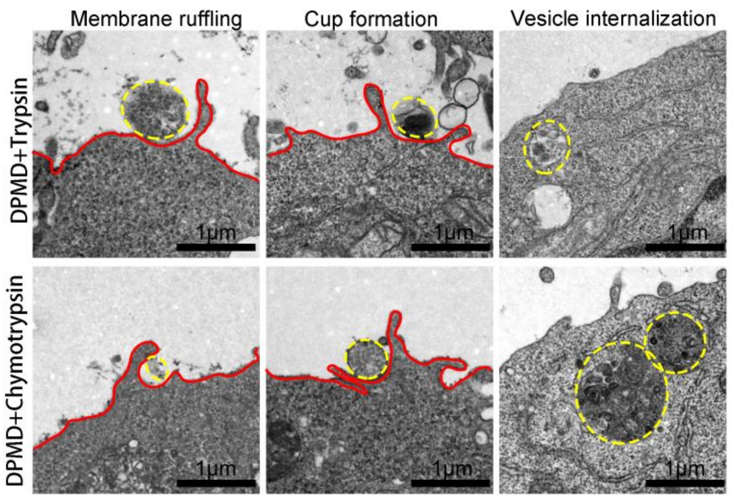

Figure S9. The TEM images of RAW264.7 macrophage cells (cell membrane: red curve) incubating with DPMD-trypsin/chymotrypsin (yellow dotted circle).

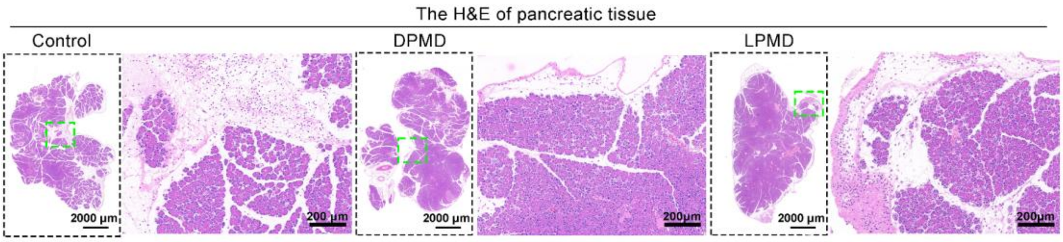

Figure S10. Typical H&E staining photograph of the pancreas three day after surgery.

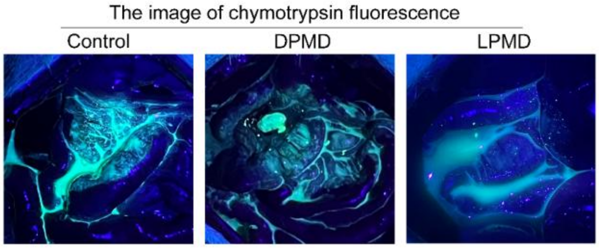

Figure S11. Images of chymotrypsin fluorescence in pancreatic common duct transection fistula model.

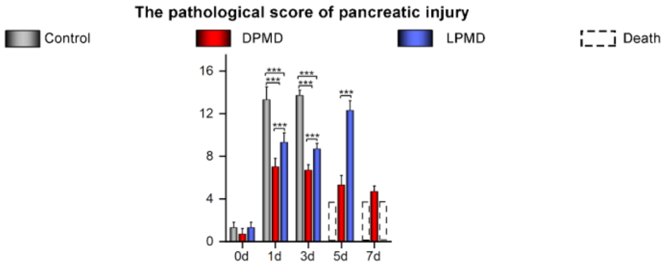

Figure S12. (**A**) The pathological score of pancreatic injury. (Data are presented as means ± SD, statistical analysis was performed using one-way ANOVA, * $p < 0.05$, ** $p < 0.01$, ***$p < 0.001$)

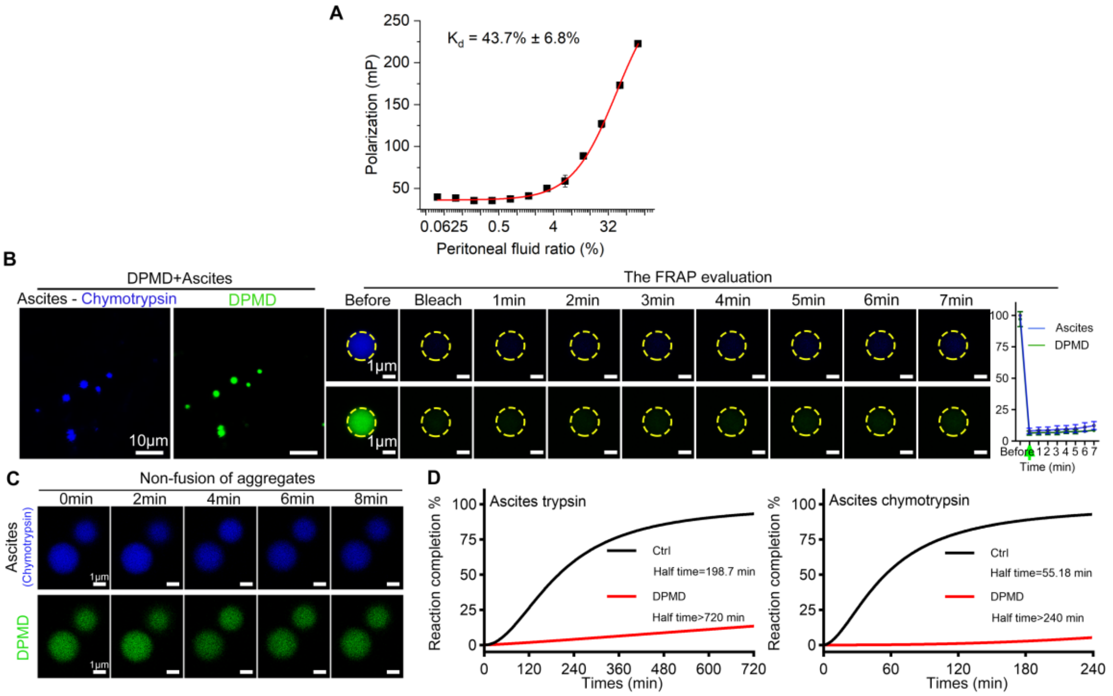

Figure S13. (**A**) The evaluation of fluorescence polarization of DPMD with different volumes ascites. (**B**) Representative time-lapse FRAP images howling the pre-bleach and recovery signals of DPMD-treated ascites^{chymotrypsin} *ex vitro*. Circles indicate the bleached area. Scale bars, 10 μm and 1 μm (zoomed-in image). (**C**) Time-lapse images of DPMD-ascites mixture aggregates, illustrating the non-fusion of two aggregates. (**D**) The inhibition evaluation of enzymes in ascites bioactivity for trypsin and chymotrypsin by DPMD.

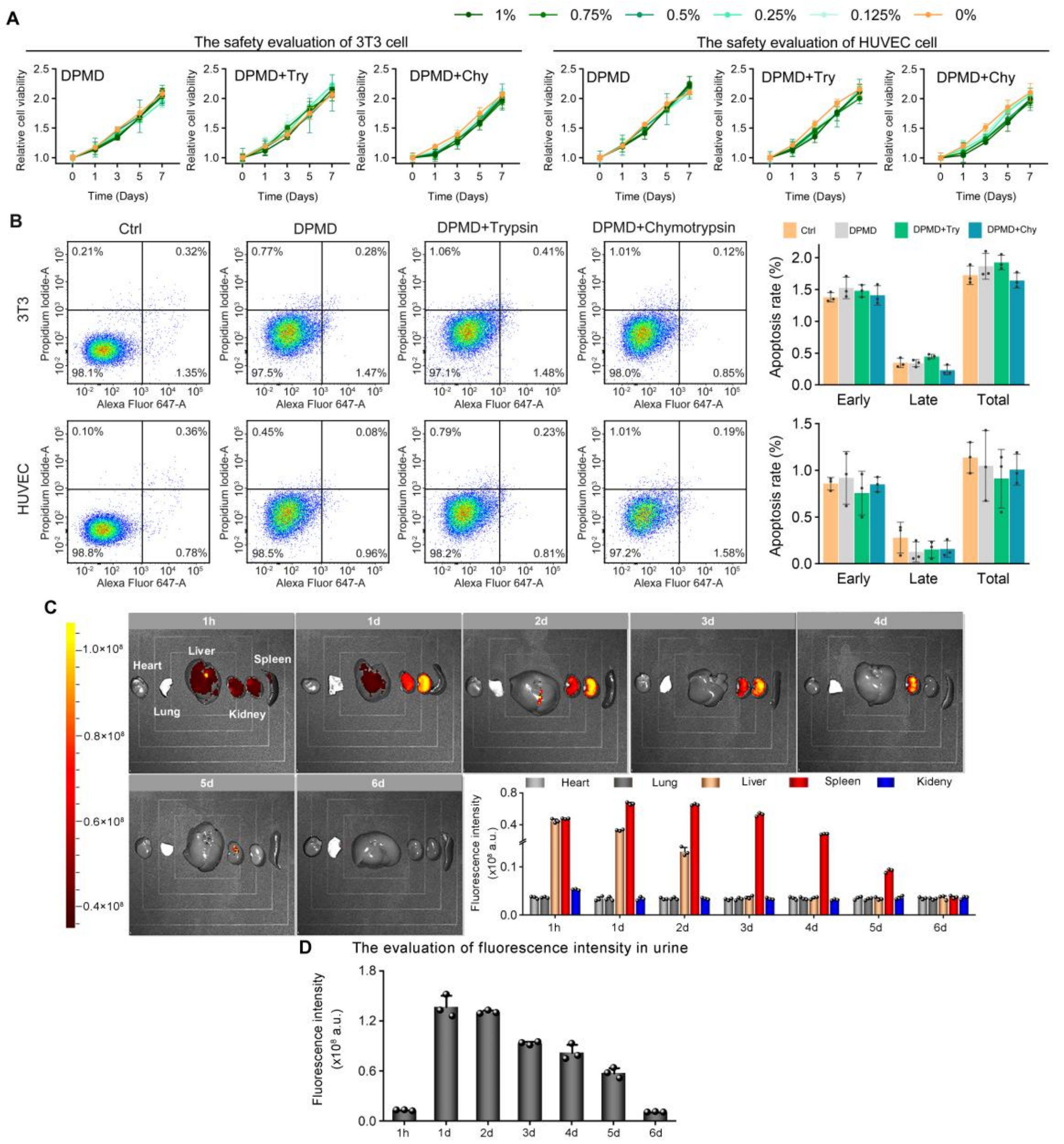

Figure S14. (**A**) The cell viability test of NIH-3T3 or HUVEC cells co-cultured with different concentrations DPMD. (**B**) The apoptosis evaluation of NIH-3T3 or HUVEC co-cultured with DPMD, DPMD & Trypsin, DPMD & Chymotrypsin by flow cytometry. (**C**) Tissue distribution and quantification of DPMD in mice organs measured by IVIS (n=3/point). The fluorescence signal from the organs after DPMD intraperitoneal injection (1 ml, 0.5%) at 1h, 2-day, 3-day, 4-day, 5-day, 6-day, 7-day. (**D**) The evaluation of distribution DMPD in urine.

3. Materials and methods

3.1 Molecular dynamics (MD) simulation

Using the GROMACS software package (version 2021.4) combined with the MARTINI force field (version 2.2) to construct a coarse-grained model of peptides. A 2000ns MD was performed on an aqueous system consisting of 30×DPMD and trypsin or chymotrypsin and Ca^{2+} ions simulation, which PDB ID are 1H4W, 1LPA and 4H4W respectively. In the initial state, peptide molecules are randomly dispersed in a cubic box of 15 × 15 × 15 nm^3 and filled with coarse-grained water. Cl^- ions and Na^+ ions are added to the box to neutralize the system charge. The cutoff for calculation of electrostatic interactions and van der Waals forces was set at 1.05 nm. The peptide group and the water/ion group were coupled to an external temperature bath using velocity, respectively. Rescaling method and pressure bath using the Parrinello-Rahman method. Update the neighbor list every 10 steps using a Verlet buffer with a cutoff distance of 1.05 nm. Trajectory analysis using Gromacs internal tools. Contact was considered formed when the minimum distance between two groups was within 0.8 nm. Pymol and VMD software were used for trajectory visualization and peptide conformation analysis.

3.2 The characterization of CD (Circular Dichroism) spectrum

CD spectra were produced by examining a 40 μM peptide sample in an aqueous solution containing 20% TFE, using a quartz cuvette with a 0.1 cm path length at room temperature. A Jasco J-810 spectropolarimeter (Japan) was used to perform the CD measurements at a scanning speed of 10 nm/min with a resolution of 0.2 nm. The raw ellipticity data was converted into molar ellipticity using the appropriate formula. A plot was made using wavelength (nm) on the x-axis to generate the

circular dichroism spectrum and molar ellipticity (kdeg·cm2/mol) on the y-axis.

3.3 Peptide Synthesis and DPMD Preparation

All peptides were synthetized according to the optimized HBTU activation/DIEA in situ neutralization protocol developed by an HBTU/HOBt protocol for FMOC-chemistry SPPS on the CS bio 336X automated peptide synthesizer. Briefly, after cleavage and deprotection in a reagent cocktail containing 88% TFA, 5% phenol, 5% H_2O and 2% TIPS, crude products were precipitated with cold ether and purified to homogeneity by preparative C18 reversed-phase HPLC. The molecular masses were ascertained by electrospray ionization mass spectrometry (ESI-MS). For DPMD preparation, peptides were reconstituted at the concentration of 5 wt% *in vivo*.

3.4 TEM

The DPMD peptide solutionfor TEM was prepared at the concentration of 0.05%w/v. When testing the reaction with the enzymes, the DPMD peptide solution wasmixed with 1% w/v trypsin and chymotrypsin solutions in a 1:l ratio, respectively. TheTEM samples were prepared by dropping the peptide solution on the copper gridSamples were stained with 2 % phosphotngstig acid solution and observed under atransmission electron microscope (Tecnai Spirit).

3.5 Ca-binding assay

$CaCl_2$ aqueous solution (Ca^{2+} concentration, 3μg/mL) and peptide aqueous solution at different concentrations (0.625-30mg/mL) were prepared using gradient dilution. Subsequently, the peptide solutions at different concentrations were mixed with 3μg/mL aqueous $CaCl_2$ solution and 20 mM sodium phosphate buffer (pH 7.4). After incubation at 37°C for 30 min, the mixture was separated by centrifugation at 4000rpm for 20 min. The supernatant was collected and filtered through a

0.22μm filter to remove residue. The calcium content of the supernatant was measured using the Calcium ion detection kit (Beyotime) according to the recommended method. The procedure was repeated four independent times.

3.6 Enzyme-peptide interactions and FRAP analysis

Peptide and enzyme solutions were prepared at varying concentrations and mixed at a 1:1 volume ratio. Solution turbidity was quantitatively assessed by monitoring absorbance at 405 nm using a BioTek spectrophotometer. For fluorescence-based characterization, peptides were labeled with FITC while enzymes were labeled with Cy5. Unless otherwise specified, both the FITC-labeled peptide solutions and Cy5-labeled enzyme solutions were prepared at a concentration of 0.5% (w/v). The high-concentration group used 1% (w/v) peptide solution. pH was adjusted using 0.1 M HCl and 1M NaOH. Fluorescent samples were imaged using a confocal microscope (Olympus FV3000). For fluorescence recovery after photobleaching (FRAP) analysis, enzyme-aggregated patches were selectively bleached at 60% laser power using 488/640 nm lasers for 10 s, followed by time-lapse imaging at 1-min intervals. This experiment was repeated three independent times. Fluorescence intensity in the photobleached region was measured using ImageJ software , with values normalized to pre-bleach baselines.

3.7 The phagocytosis assays

A phagocytosis assay was performed using the murine macrophage cell line RAW 264.7. The cell line was maintained at 37 °C with 5% CO_2 in DMEM culture medium supplemented with 10% FBS. After 24 hours of routine culture, the cells in different groups were cultured in fresh medium supplemented with Amiloride (1 mM) or Cytochalasin D (10 μM) for another 2 hours, respectively.

Then, cells were co-incubated with Cy5-labeled proteins (trypsin^{Cy5} and chymotrypsin^{Cy5}) and DPMD treated proteins Cy5 for 30 min. Subsequently, cells were washed thrice with PBS, fixed with 4% paraformaldehyde, and the nucleus was dyed with DAPI. Observations were performed using a confocal microscope (Olympus, FV3000). This experiment was repeated three independent times. The mean fluorescence intensity was measured using Image J software. The fluorescence values were calculated after background subtraction (fluorescence intensity of regions without cells).

3.8 Measurements of ThT fluorescence

The 0.5% (w/v) aqueous peptide solutions, enzyme solutions, and peptide-enzyme mixtures were supplemented with thioflavin T (ThT, 100 μM, Aladdin). ThT Fluorescence assay was measured on a Steady-state and lifetime Fluorescence Spectrometer (FLS1000). A filter with an excitation wavelength of 440nm and an emission wavelength of 450~700 nm was used. All experiments were conducted at 25.0°C, with triplicate independent measurements performed for each sample.

3.9 Enzyme activity assay

The trypsin and chymotrypsin were sourced from Diamond and Aladdin companies, respectively. The activity detection experiment was conducted according to the optimized method in the kit manual. In the experimental group, DPMD was incorporated into trypsin. The final concentration of trypsin was set at 62.5 μg/mL, and the final concentration of DPMD was 5 mg/mL. In the corresponding control group, an equal volume of the buffer solution prepared by the kit was added. Subsequently, the enzyme-peptide mixture was added to the reaction system containing N-Benzoyl-L-Arginine-Ethylester (BAEE) at a ratio of 1:100 (where the proportion of the enzyme-peptide mixture solution was 1) for the detection of enzyme activity. The reaction was carried out at 37 °C,

and the product's absorbance at 253nm was dynamically and real-time monitored using a microplate reader.

During the chymotrypsin activity assay, DPMD was incorporated into chymotrypsin. The final concentration of chymotrypsin was set at 10 μg/mL, and the final concentration of DPMD was 5 mg/mL. In the corresponding control group, an equal volume of the buffer solution prepared by the kit was added. Subsequently, the enzyme-peptide mixture was added to the reaction system containing the substrate N-Benzoyl-L-Tyrosine Ethyl Ester (BTEE) at a ratio of 1:100 (where the proportion of the enzyme-peptide mixture solution was 1) for the detection of enzyme activity. The reaction was carried out at 25 °C, and the product's absorbance at 256 nm was dynamically and real-time monitored using a microplate reader. The experimental result was represented by the enzyme reaction's degree of completion. All the tests were repeated three independent times.

3.10 CCK8 assay

NIH-3T3 cells were obtained from the National Collection of Authenticated Cell Cultures. HUVECs were obtained from Procell. All cells were maintained in DMEM media at 37°C with 5% CO_2. For the assay, 800 cells/well were seeded into 96-well plates and treated with serial dilutions of DPMD and enzymes (trypsin and chymotrypsin). Cell viability was evaluated by the CCK-8 assay (Dojindo) at 1-, 3-, 5-, and 7-days post-treatment. Briefly, 10 μL of CCK-8 reagent was added to 100 μL of culture medium per well. The absorbance at 450 nm was recorded using a microplate reader (BioTek).

3.11 Apoptosis Assay

Cells were treated with DPMD and enzymes (trypsin or chymotrypsin) for 48 h. The apoptosis assay

was performed using an Annexin V-Alexa Fluor 647/PI kit (Solarbio) according to the manufacturer's protocol. Briefly, adherent cells were detached with 0.25% EDTA-free trypsin, combined with floating cells from the supernatant. The pellet was washed twice with cold PBS and resuspended in 100 μL of 1× binding buffer. After adding Annexin V-647 and PI, cells were incubated for 15 min in the dark at room temperature. Samples were analyzed within 1h on a Sony MA900 flow cytometer.

3.12 Animal fluorescence imaging

Rats were intraperitoneally injected with 1 mL of rhodamine-labeled 0.5% (w/v) DPMD. At the predetermined time points post-injection (1h, 1d, 2d, 3d, 4d, 5d, 6d), the rats were sacrificed, and the major organs (including heart, liver, spleen, lungs, and kidney) were harvested. The fluorescence emission intensity of the organs was measured using IVIS Spectrum system (PerkinElmer). The excitation filter wavelength was set at 561nm, and the emission filter wavelength was set at 572 nm.

3.13 Construction of a rat pancreatic splenic duct transection model

Animal studies were performed according to the protocols approved by the Institution Guidelines and were approved by the Laboratory Animal Center of Xijing Hospital of The Fourth Military Medical University (No. IACUC-20210517). ALL rats were purchased from the Laboratory Animal Center of The Fourth Military Medical University. The mice were housed under standard specific pathogen-free conditions with a 12h–12h light–dark cycle.

The male SD rats weighing 250-300g were selected. The day before modelling, prepare the abdominal skin of the rats. On the day of modelling, induce and maintain anesthesia in the rats using isoflurane (RWD Life Science Co., Ltd.). After making a midline incision in the abdomen and separating the gastroduodenal ligament, locate the pancreatic duct in the head of the pancreas.

Without damaging the surrounding pancreatic tissue and capillaries, a transverse incision on the pancreatic splenic duct was performed using micro-scissors. Successful modelling is confirmed by observing external leakage of pancreatic fluid. Place a self-made drainage tube into the rat's abdominal cavity with its outlet positioned at the site of the transected splenic duct, then exit through the abdominal wall and fix it there.

3.14 Construction of rat pancreatic common duct transection model

Animal studies were performed according to the protocols approved by the Institution Guidelines and were approved by the Laboratory Animal Center of Xijing Hospital of The Fourth Military Medical University (No. IACUC-20220308). ALL rats were purchased from the Laboratory Animal Center of The Fourth Military Medical University. The rats were housed under standard specific pathogen-free conditions with a 12h–12h light–dark cycle.

The preoperative preparation and postoperative care for the rat model were consistent with those of the rat pancreatic splenic duct transection pancreatic fistula model, except for the transecting of the pancreatic common duct of the pancreas in modeling. A custom-made drainage tube outlet was positioned around the area where the pancreatic common duct was transected.

3.15 Evaluation of the effect of DPMD on pancreatic duct transection model

The male SD rats were randomly assigned to six rats in each group. Prepare a pancreatic duct transection model with a pancreatic fistula and apply 0.3 mL of normal saline, 0.3 ml of DPMD, and 0.3 mL of LPMD at the incision site. After 3days, administer 3 mL of MeO-Succ-Arg-Pro-Tyr-AMO solution (ATT Bioquest) to the pancreatic stump and surrounding tissues for fluorescence imaging in real-time under dark field conditions while using an API filter. Blood and ascitic fluid

samples were collected on the day of surgery and postoperative days 1, 3, 5, and 7. Tissue samples of the pancreatic splenic duct transection model were collected on the first postoperative day, and tissue samples of the pancreatic common duct transection model were collected on the third postoperative day. Peritoneal fluid and serum were collected via retro-orbital vein puncture and analyzed for amylase and lipase using an ELISA kit (BeyoClick), according to the kit manual. Inflammatory factors in serum were assessed using Luminex technology.

The basal ascites amylase level (the ascites amylase level on day 0) was used as the baseline value to evaluate the degree of pancreatic fistula. We define the amylase concentrations greater than three times the baseline value as pancreatic fistula, significantly less than twice the baseline as not leak pancreatic fistula, greater than twice the baseline definition, but less than three times the baseline definition as pancreatic fistula or mild pancreatic fistula.

3.16 H&E staining

The tissue samples were collected and fixed with 4% paraformaldehyde. The paraffin-embedded tissue sections were sectioned into 4 μm thick slices and used for Haematoxylin-Eosin staining. All slices were photo-captured by a fully automatic slice scanning system (Pannoramic).

3.17 Fluorescence polarization

Using the solid-phase peptide synthesis (SPPS) method, the N-terminal cysteine of the peptide DPMD was labeled with fluorescein isothiocyanate. The labeled peptide was purified by preparative C4 reversed-phase high-performance liquid chromatography (RP-HPLC) and lyophilized. Fluorescence polarization experiments with DPMD and trypsin and chymotrypsin were conducted in Corning® 96-well black plates. A SpectraMax M5 multi-mode microplate reader was used for

data acquisition. A series of enzyme solutions with varying concentrations (maximum concentrations of trypsin and chymotrypsin were 20 μM each) were prepared in Tris-HCl buffer (10 mM Tris, 150 mM NaCl, 1 mM EDTA, pH 7.0). These enzyme solutions were added to the 96-well plate and incubated with 100 nM DPMD at room temperature for 30 minutes, with triplicates. Fluorescence polarization was measured with an excitation wavelength of 470 nm and an emission wavelength of 530 nm.

Control experiments using using both a non-target protein of similar size and abundance, as well as binding-pocket sealed with inhibitor of trypsin and chymotrypsin. Trypsin/Chymotrypsin was saturated and blocked by Aprotinin (Yeasen Biotechnology, trypsin/chymotrypsin inhibitor). Both inhibitor and protein were used at 20 μM. The highest peritoneal fluid concentration was the undiluted stock.

Plot the fluorescence intensity versus concentration data as scatter plots using Origin software. For data analysis, fit the concentration-fluorescence polarization data with the logistic function in Origin 2021 software to obtain the fitting curve and determine the half-maximal effective concentration (EC50). The calculation formula for K_d value is:

$$K_d = EC_{50} - \frac{c_{DPMD}}{2}$$

where c_{DPMD} is the concentration of peptide DPMD, i.e., 100 nM….

3.18 Statistical Analysis

All results and data were expressed with mean ± standard deviation (S.D.). Student t-tests was used for statistical significance analysis to determine the significance of two individual data. A probability value of 95 % ($p < 0.05$, denoted by * in figures), 99 % ($p < 0.01$, denoted by ** in figures) and 99.99 % ($p < 0.001$, denoted by *** in figures) were used to compare the significance

across each group.
